# Multi-Objective Optimization for Data Center HVAC Systems Based on Edge–Cloud Collaborative Deep Reinforcement Learning

**DOI:** 10.3390/s26165031

**Published:** 2026-08-07

**Authors:** Shichao Huang, Yibing Zhou, Yuan Liu

**Affiliations:** 1 School of Electronic and Information Engineering, South China University of Technology, Guangzhou 510000, China; eehuangshichao@mail.scut.edu.cn (S.H.); 202421011976@mail.scut.edu.cn (Y.Z.); 2 Guangzhou Power Supply Bureau, Guangzhou 510000, China

**Keywords:** edge–cloud collaboration, deep reinforcement learning, HVAC systems, multi-objective optimization

## Abstract

The sustained growth of cloud computing and AI training workloads drives data center expansion. Optimizing their control is therefore critical for reducing operational costs. Edge real-time control is indispensable for guaranteeing thermal safety, data sovereignty, and offline availability. Yet deploying Deep Reinforcement Learning (DRL) in production Heating, Ventilation, and Air Conditioning (HVAC) environments confronts cold-start risks, edge–cloud computational asymmetry, and multi-objective conflicts spanning energy efficiency, electricity cost, and thermal safety. To address these challenges, this paper proposes an edge-cloud collaborative physics-informed reinforcement learning framework for production data center HVAC control. The framework integrates a physics-informed cold-start solution using Adaptive Particle Swarm Optimization (APSO) to generate physically constrained initial policies on a gray-box digital twin without expert demonstration data, a three-time-scale edge–cloud architecture coordinating minute-level edge Soft Actor-Critic (SAC) real-time inference, weekly edge APSO online model identification, daily cloud Non-dominated Sorting Genetic Algorithm III (NSGA-III) thermal storage scheduling, and a constraint-aware safe projection layer that embeds thermal safety hard constraints directly into the neural network policy. The framework is validated through a seven-month production deployment spanning the complete summer-to-winter transition, comprising approximately 3.2 million sensor records and evaluated with rigorous statistical methods.

## 1. Introduction

The rapid growth of cloud computing and AI training workloads has driven data center expansion. Energy consumption has become the dominant component of operational costs. The International Energy Agency (IEA) projects that global data center electricity consumption will exceed 1000TWh by 2026. Heating, Ventilation, and Air Conditioning (HVAC) cooling systems typically account for 30% to 45% of total facility power usage [1]. Reducing cooling energy while guaranteeing thermal safety is a practical problem that demands engineering solutions.

HVAC control must execute at the edge rather than merely serving as an optional deployment alternative. This necessity stems from four unavoidable engineering constraints. (1) Latency sensitivity: sudden weather changes demand second-level responses. Cloud round-trip delays, also on the order of seconds, can trigger chiller surge alarms and emergency shutdowns. (2) Physical safety: chilled water supply temperatures below 7 °C cause condensation on fan coil units, damaging server hardware. Real-time protection at the edge is indispensable. (3) Data sovereignty: hundreds of sensors sampling continuously at minute-level intervals generate massive raw data streams. Edge-side pre-processing substantially reduces uplink bandwidth requirements [2,3,4]. *(4) Offline availability*: edge nodes must autonomously maintain control during network jitter and disconnections. Although cloud latency can be partially mitigated through connection pooling and pre-fetch caching, empirical comparisons demonstrate that edge deployment retains irreplaceable advantages in control-period compliance and offline availability.

Deep Reinforcement Learning (DRL) has demonstrated advantages in sequential decision-making for building energy systems [5,6,7]. Unlike Model Predictive Control (MPC), which requires explicit system models, DRL agents learn optimal state–action mapping policies through sustained interaction with the environment [8]. Recent advances have further embedded thermodynamic constraints into policy networks [9] and introduced safe exploration mechanisms [10,11]. However, deploying DRL in production HVAC environments faces three coupled challenges: (i) the cold-start problem, where randomly initialized policies risk thermal safety incidents during exploration; (ii) computational asymmetry between cloud-grade training and edge-constrained inference [12,13]; and (iii) multi-objective conflicts spanning energy efficiency, electricity cost, and thermal safety.

The cold-start problem constitutes the most critical barrier to production deployment. During the initial exploratory phase, policy outputs are nearly random, and thousands of environment interactions are typically required before usable control laws emerge [14]. Conventional cold-start solutions include Behavior Cloning (BC), which depends on expert demonstration data that data centers typically lack, and Offline RL, which remains sensitive to distribution shift when deployed online [15]. Model-based pre-training using MPC-generated trajectories can accelerate convergence, yet it relies on calibrated thermodynamic models that are rarely available in production data centers [8]. Recent studies have explored physics-informed approaches to inject domain knowledge into DRL. Wang et al. [16] integrated computational fluid dynamics into a safe RL framework for data center cooling. Cao et al. [17] employed a Lyapunov-based constrained formulation for safe exploration. Yu et al. [18] proposed a linear-program-based safe projection layer for district cooling systems, and Jin et al. [19] introduced statewise projection via control barrier functions for constrained RL. Despite these advances, existing cold-start solutions share two structural limitations. First, they depend on either expert demonstration data or precise prior models. Second, they omit online model recalibration: the performance of chillers, pumps, and heat exchangers degrades continuously due to fouling, refrigerant leakage, and mechanical wear, causing fixed-parameter models to progressively lose accuracy. A cold-start paradigm that requires neither expert data nor a precise prior model, while enabling periodic recalibration through online identification, remains an open problem.

Beyond the cold-start challenge, the integration of real-time control, online model identification, and day-ahead scheduling in HVAC systems has received limited attention. Existing studies typically address these subproblems in isolation. Cui and Liu [20] combined SAC with hybrid search for multi-zone ventilation control. Zhang et al. [21] proposed a DRL framework integrated with whole-building energy models. Jang et al. [22] developed active RL for robust building control under extreme weather. These works treat model identification, real-time control, and thermal storage scheduling as decoupled tasks. The fundamental gap is that no prior work has established a sequential integration mechanism among these three computational modules—one in which physics-based pre-training initializes the control policy, online identification periodically recalibrates the physics model using operational data, and day-ahead scheduling produces thermal storage plans that are dispatched to the edge for coordinated execution with real-time control. Without such integration, each module operates on stale or locally optimal information.

The majority of existing DRL–HVAC studies rely on simulation validation or short-term pilot tests [5]. Although Zhan et al. [15] reported 2000 hours of production deployment for offline RL in data center cooling, multi-seasonal validation spanning the full transition from summer to winter under a coupled optimization framework remains extremely scarce. Public benchmarks such as CityLearn [23], SustainCluster and ASHRAE GEP III [24] provide valuable references but do not substitute for production-grade empirical studies under coupled multi-objective optimization.

To address these challenges, we propose an edge–cloud collaborative physics-informed reinforcement learning framework for production data center HVAC control. The framework integrates physics-based pre-training, edge-resident real-time inference, and cloud-based day-ahead scheduling into a coordinated system validated through long-term production deployment. The principal contributions are as follows.

First, we provide a production-grade empirical validation of DRL-based HVAC control spanning seven months (210 days) across the complete summer-to-winter transition, comprising approximately 3.2 million sensor records sampled at 3 min intervals. Recent surveys have identified the absence of such long-term, real-operational-data validation as a critical research gap [5]. Unlike prior studies that rely on simulation or short-term pilot tests, our evaluation employs rigorous statistical methods: Wilcoxon signed-rank tests with Bonferroni correction ($\alpha^{*}=0.0071$), Newey–West heteroskedasticity-autocorrelation consistent standard errors, and Bootstrap confidence intervals based on 10,000 resampling repetitions. The proposed framework achieves a 17.8% cooling cost reduction (95% CI: [16.1%,19.5%]) with zero thermal violations and Cohen’s d=2.88 relative to the PID baseline.

Second, we propose a physics-informed cold-start engineering solution that eliminates the dependence on expert demonstration data and calibrated high-fidelity models. An Adaptive Particle Swarm Optimization (APSO) algorithm searches for optimal control setpoints on a gray-box physics-based digital twin across a broad spectrum of representative operating conditions. The resulting state–action pairs pre-train the Soft Actor-Critic (SAC) policy network through supervised learning, providing physically constrained initialization. Unlike BC or MPC-trajectory-based pre-training, this approach requires neither expert data nor a calibrated high-fidelity model. Weekly online APSO identification recalibrates the thirteen gray-box model parameters using measured operational data, enabling periodic model recalibration to accommodate equipment drift. Production results demonstrate that this solution achieves a 3.7× convergence speedup and a 12.5% cooling cost reduction on the first deployment day, with zero thermal violations during the initial week.

Third, we design a three-time-scale edge–cloud architecture and a constraint-aware safe projection layer. The architecture coordinates minute-level edge SAC inference, weekly edge APSO model identification, and daily cloud Non-dominated Sorting Genetic Algorithm III (NSGA-III) thermal storage scheduling, with operational data from the edge feeding back to support model recalibration. The safe projection layer enforces hard thermal constraints through a smooth, monotonic Sigmoid mapping that guarantees output boundedness by construction, structurally eliminating out-of-bound actions unlike penalty-based methods [18,19]. Ablation experiments confirm that removing any single module produces statistically significant performance degradation (p<0.0071, Cohen’s *d* from 0.68 to 2.45), and removing the projection layer produces three thermal violation events versus zero in the full system.

## 2. Related Work

**DRL for HVAC control.** Edge computing has emerged as a critical enabler for smart building applications. Shi et al. [2] identified latency-sensitive IoT applications as the primary use case for edge intelligence. Chen and Ran [25] systematically reviewed deep learning architectures for edge computing, demonstrating that edge inference can reduce latency by up to 80% for building monitoring tasks. Su et al. [26] proposed a distributed optimal control strategy for HVAC systems adopting edge computing, experimentally validating real-time control as a high-impact application for building energy management. Vazquez-Canteli and Nagy [5] surveyed reinforcement learning methods for demand response. Zhang et al. [21] proposed a DRL framework integrating whole-building energy models. Chen et al. [27] introduced Gnu-RL, embedding thermodynamic constraints into neural network architectures. Wang et al. [16] developed a physics-informed safe RL framework for data centers. Cai and Liu [20] proposed a hierarchical HVAC control method combining SAC with hybrid search. Hedayat et al. [9] embedded thermodynamic constraints directly into the policy network of a continuous control agent. Although Zhan et al. [15] reported 2000 hours of production deployment for offline RL in data center cooling, none of these works has achieved multi-season empirical validation under a coupled multi-objective optimization framework.

**RL cold start.** When deploying DRL in production HVAC systems, cold start constitutes an unavoidable challenge. During the initial exploratory phase, policy outputs are nearly random, followed by severe fluctuations in chilled water temperature and frequent equipment cycling. Severe cases can even cause thermal safety incidents. The core of the cold-start problem lies in the DRL agent’s lack of prior knowledge: thousands to tens of thousands of environmental interactions are typically required before basic control laws are learned. Several recent studies have explored model-based DRL pre-training for building control. Drgona et al. [8] systematically surveyed building control frameworks combining MPC with data-driven methods. Pre-training DRL agents using MPC-generated trajectories can accelerate convergence but depend on calibrated thermodynamic models. Levine et al. [14] summarized the offline reinforcement learning paradigm, which uses physical simulation or historical data to generate offline datasets for batch RL pre-training. Zhan et al. [15] extended this line with a physics-informed offline RL framework deployed in a production data center, yet their approach still relies on a pre-collected offline dataset and does not incorporate online model recalibration. Cao et al. [17] and Wang et al. [16] explored safe RL for data center cooling using Lyapunov-based constraints and physics-guided models, respectively. Yu et al. [18] and Jin et al. [19] proposed projection-based safe layers for constrained RL in cooling and general control domains. Wang et al. [11] and Esmaeili et al. [10] further advanced safe DRL for building energy management. These methods share two common limitations. First, they rely on precise prior models or pre-collected datasets, which are rarely available in production data centers. Second, they omit online model recalibration to track equipment degradation.

## 3. Methodology

**Figure 1:**
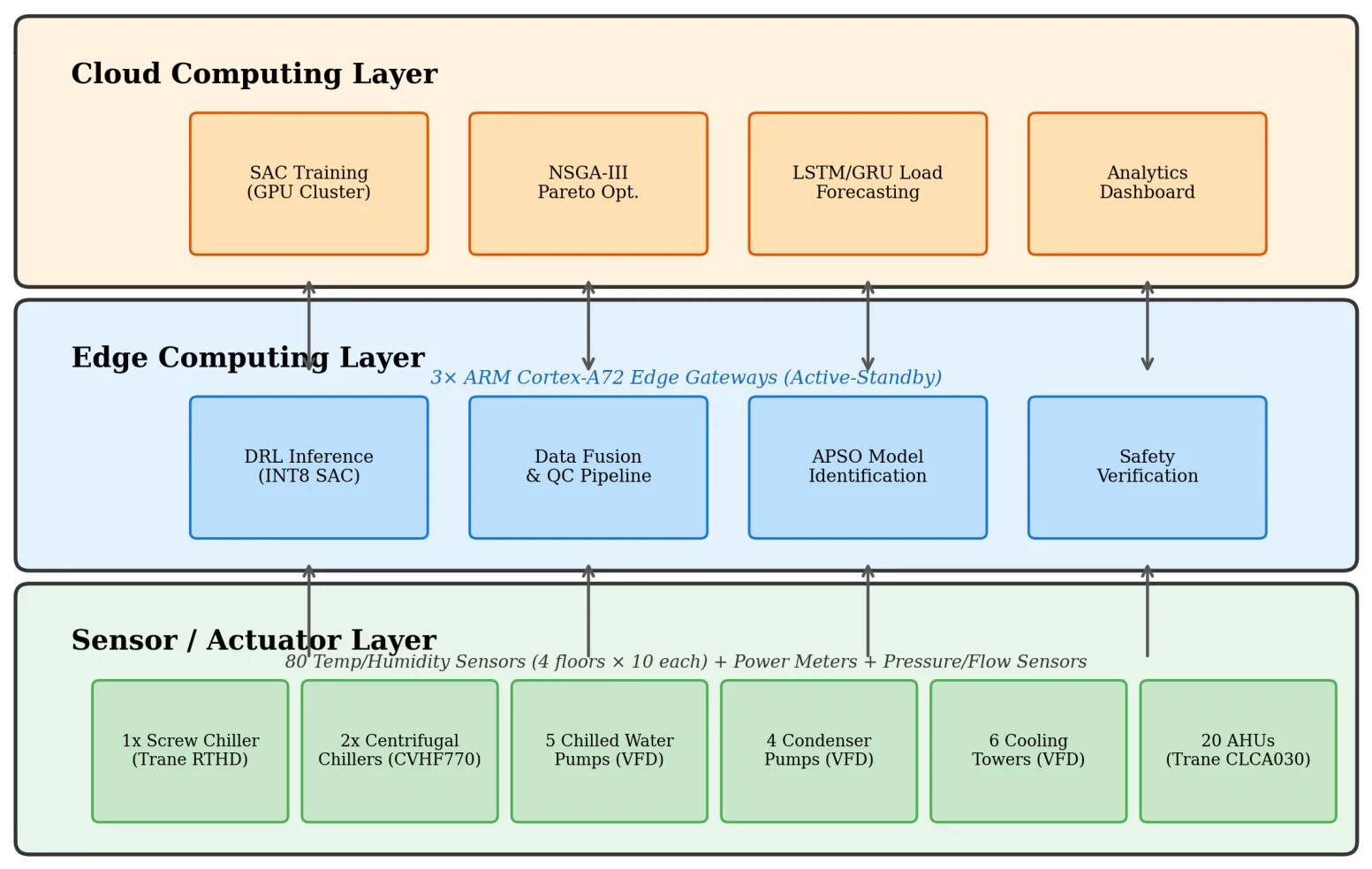
System Architecture for edge–cloud collaborative data center HVAC optimization.

**Figure 2:**
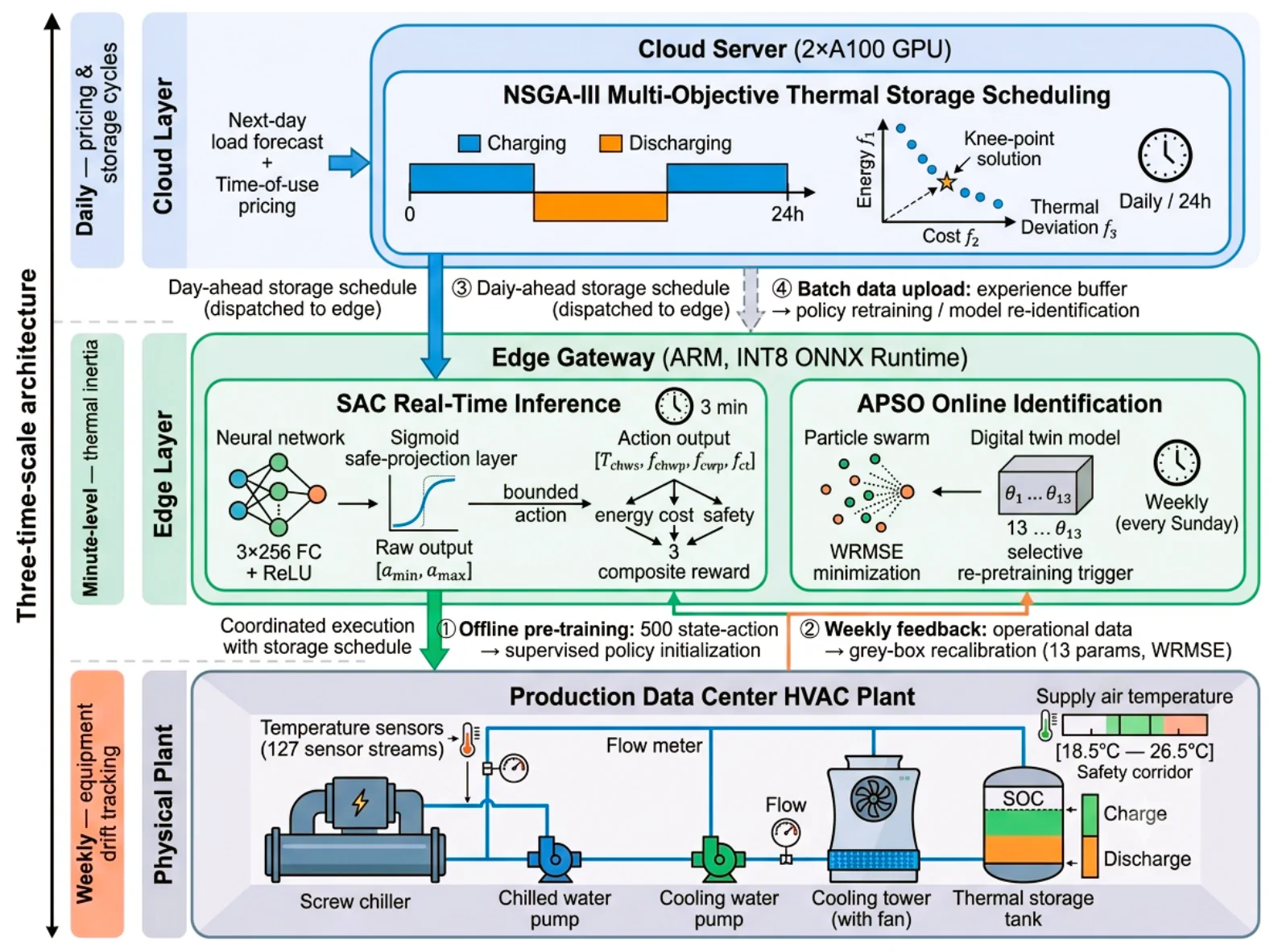
Three-layer hierarchical control architecture.

In this study, we propose an edge–cloud collaborative physics-informed reinforcement learning control framework for data center Heating, Ventilation, and Air Conditioning (HVAC) systems. It addresses the coupled optimization problem among high-frequency real-time control, day-ahead energy scheduling, and hard safety constraints of equipment. As shown in Figure 1, the framework comprises three core computational modules: a physics model pre-training module based on adaptive particle swarm optimization, an edge-resident deep reinforcement learning inference module, and a cloud-based multi-objective cold storage scheduling optimization module. These three modules form a coordinated control system comprising offline pre-training, online edge inference, and cloud-based day-ahead optimization, with operational data from the edge feeding back to support weekly model recalibration. The information flow and control logic are illustrated in Figure 2.

During the offline pre-training phase, the system does not interact with the real physical environment. Instead, it uses an identified gray-box physics-based digital twin model and adaptive particle swarm optimization algorithm to search for optimal control setpoints across a wide range of representative operating conditions. This process constructs high-quality state–action data pairs, which are then used to pre-train the policy network through supervised learning. The resulting initial weight files are deployed and loaded onto edge devices.

In the online operation phase, the edge controller operates on a three-minute control cycle. It performs sensor data reading, state vector construction, online standardization, policy network forward inference, constraint-aware safety projection, and control command issuance. The controller also maintains a local experience replay buffer and periodically transmits data back to the cloud.

For the day-ahead scheduling phase, the cloud server leverages hourly load forecasts and time-of-use electricity pricing information for the next day. It conducts three-objective Pareto optimization of charging and discharging power for the cold storage system at a 24 h time granularity. A knee point solution is selected from the Pareto front as the next day’s scheduling plan and sent to the edge controller, enabling coordinated optimization of cold storage during off-peak hours and cold discharge during peak hours.

### 3.1. Problem Formulation

We formulate the optimal control problem for the HVAC system as a discrete-time Markov Decision Process (MDP), denoted by the quintuple (S,𝒜,𝒫,r,γ). Here, S represents the state space, 𝒜 the action space, $𝒫(s_{t+1}|s_{t},a_{t})$ the state transition probability, $r(s_{t},a_{t})$ the immediate reward function, and γ∈[0,1) the discount factor. This formulation reframes the data center cooling control problem as a sequential decision-making task, enabling reinforcement learning algorithms to optimize the long-term cumulative return directly.

The state vector $s_{t}\in R^{38}$ comprises seven semantic feature groups. These encompass meteorological conditions, load profiles, equipment status, electricity pricing, water system states, floor-level environmental measurements, and temporal encodings. Specifically, the meteorological group includes the outdoor dry-bulb temperature $T_{\mathrm{out}}$, the outdoor relative humidity $\phi_{\mathrm{out}}$, and the outdoor wet-bulb temperature $T_{\mathrm{wb}}$. The load group contains the real-time cooling load $Q_{\mathrm{load}}$, the partial load ratio (PLR), moving averages and standard deviations of the cooling load, a rolling mean of the outdoor temperature, and several placeholder dimensions. The chiller status group captures the chilled water supply and return temperatures, together with the cooling water supply and return temperatures. The pricing group incorporates the real-time electricity price $p_{\mathrm{price}}^{t}$ and a peak-period indicator. The water system group records the chilled and cooling water flow rates, along with differential pressure readings. The floor-level environmental group comprises the supply air temperature, return air temperature, and relative humidity for each floor. The temporal encoding group includes the normalized control step index t/480, supplemented by sinusoidal and cosinusoidal periodic encodings sin(2πt/480) and cos(2πt/480), where *t* denotes the current control step index.

In the production environment, the state dimensionality derives from one hundred and twenty-seven sensor streams processed through feature engineering and dimensionality reduction. Within the experimental demonstration system, missing dimensions are padded with sliding statistics or constant placeholders to maintain dimensional consistency across the state vector.

The continuous action vector $a_{t}\in R^{4}$ encodes four control variables. The physical meaning of each dimension and the feasible region boundaries follow:

(1) $$a_{t}={\left[\begin{array}{cccc} T_{\mathrm{chws}} & f_{\mathrm{chwp}} & f_{\mathrm{cwp}} & f_{\mathrm{ct}} \end{array}\right]}^{⊤}.$$

Here, $T_{\mathrm{chws}}$ denotes the chilled water supply temperature in degrees Celsius; $f_{\mathrm{chwp}}$ the chilled water pump frequency in Hertz; $f_{\mathrm{cwp}}$ the cooling water pump frequency in Hertz; and $f_{\mathrm{ct}}$ the cooling tower fan frequency in Hertz. Lower and upper bound vectors, $a_{\min}$ and $a_{\max}$, define the action feasible set 𝒜:

(2) $$\begin{array}{cc} 𝒜 & =\left\{a\in R^{4}|a_{\min}⪯a⪯a_{\max}\right\},\\ a_{\min} & ={\left[\begin{array}{cccc} 7.0 & 25.0 & 25.0 & 15.0 \end{array}\right]}^{⊤},\\ a_{\max} & ={\left[\begin{array}{cccc} 14.0 & 50.0 & 50.0 & 50.0 \end{array}\right]}^{⊤}. \end{array}$$

A composite reward function balances energy efficiency optimization, electricity cost reduction, and thermal safety constraints:

(3) $$r\left(s_{t},a_{t}\right)=\omega_{1}r_{\mathrm{energy}}+\omega_{2}r_{\mathrm{cost}}+r_{\mathrm{safety}},$$

where $\omega_{1}$ and $\omega_{2}$ denote dynamic weighting coefficients. These weights are adjusted online according to the time of day: during peak-price hours, $\omega_{2}$ is increased to prioritize cost reduction; during off-peak hours, $\omega_{1}$ is increased to prioritize energy efficiency. The baseline power $P_{\mathrm{base}}$ is defined as the daily average electrical power of Scheme A (PID baseline), and the baseline cost $C_{\mathrm{base}}$ as the corresponding daily electricity cost. The energy efficiency term $r_{\mathrm{energy}}$ is defined as the negative ratio of the total system electrical power $P(s_{t},a_{t})$ to the baseline power $P_{\mathrm{base}}$:

(4) $$r_{\mathrm{energy}}=-\frac{P(s_{t},a_{t})}{P_{\mathrm{base}}}.$$

The electricity cost term $r_{\mathrm{cost}}$ is defined as the negative ratio of the single-period electricity cost to the baseline cost $C_{\mathrm{base}}$, scaled by twenty-four to achieve daily-level calibration:

(5) $$r_{\mathrm{cost}}=-\frac{P\left(s_{t},a_{t}\right)\cdot p_{\mathrm{price}}^{t}\cdot \tau }{C_{\mathrm{base}}\cdot 24}.$$

In this expression, τ=3/60 h represents the control interval duration, and $p_{\mathrm{price}}^{t}$ denotes the real-time electricity price during the *t*-th period, measured in Yuan per kilowatt-hour. The thermal safety penalty term $r_{\mathrm{safety}}$ adopts a quadratic form, imposing severe penalties whenever the supply air temperature $T_{\sup}$ deviates from the prescribed safety corridor:

(6) $$r_{\mathrm{safety}}=-\lambda_{h}\max{\left(0,T_{\sup}-\bar{T}\right)}^{2}-\lambda_{l}\max{\left(0,\underline{T}-T_{\sup}\right)}^{2},$$

where $\bar{T}=26.5\;{}^{\circ}C$ indicates the upper thermal safety bound, $\underline{T}=18.5\;{}^{\circ}C$ the lower bound, and $\lambda_{h}$, $\lambda_{l}$ the penalty coefficients. These bounds incorporate a 0.5 °C safety margin relative to the hard physical limits of 27.0 °C and 18.0 °C, respectively. This reward structure compels the policy to optimize energy efficiency while penalizing every temperature violation quadratically, confining the supply air temperature strictly within the safety corridor.

A physics-based model computes the total system electrical power $P(s_{t},a_{t})$, incorporating the chiller coefficient of performance, pump affinity laws, and the cooling tower power consumption model. Its explicit formulation will be presented in the following subsection.

### 3.2. Physics-Based Model Pre-Training and Policy Initialization via Adaptive Particle Swarm Optimization

To circumvent the safety risks and sampling costs associated with direct online interaction between reinforcement learning and the physical plant, we introduce an offline pre-training stage prior to deployment. This stage leverages a physics-based digital twin model. Two core steps constitute this phase. An adaptive particle swarm optimization (APSO) algorithm searches for optimal control setpoints across a broad spectrum of representative operating conditions on the identified physics-based twin. Subsequently, the policy network undergoes supervised pre-training to acquire a reasonable initial policy distribution.

#### 3.2.1. Gray-Box Physics-Based Digital Twin Model

The digital twin adopts a gray-box structure that combines first-principles thermodynamic formulations with data-driven parameter identification. Unlike white-box models that require detailed geometric and material specifications of every component—information typically unavailable in operational data centers—the gray-box approach retains the causal structure of physical laws (e.g., pump affinity laws, heat transfer correlations) while treating a set of aggregate coefficients as identifiable parameters. These parameters are initialized from manufacturer nameplate data and subsequently recalibrated using measured operational data through APSO online identification. This design ensures that the model remains physically interpretable while adapting to site-specific conditions and progressive equipment drift.

The model comprises thirteen identifiable parameters partitioned across three sub-models: six for the chiller, four for the water pumps, and three for the cooling tower. Table 1 summarizes the parameter vector $\theta ={\left[\theta_{1},\ldots ,\theta_{13}\right]}^{⊤}$, their physical meanings, initial values derived from nameplate data, and physically feasible search bounds.

The chiller coefficient of performance (COP) is modeled using a simplified Gordon–Ng formulation that captures three dominant physical dependencies: the COP increases with higher chilled water supply temperature (reduced lift between evaporator and condenser), decreases with higher wet-bulb temperature (elevated condensing pressure), and degrades under low part-load conditions (cycle losses and standby heat gain). The model is expressed as:

(7) $$\mathrm{COP}\left(T_{\mathrm{chws}},T_{\mathrm{wb}},\mathrm{PLR}\right)=\underset{\mathrm{base}\;\mathrm{COP}\;\mathrm{at}\;\mathrm{full}\;\mathrm{load}}{\underset{︸}{\left[a_{0}+a_{1}\left(T_{\mathrm{chws}}-7\right)-0.10\left(T_{\mathrm{wb}}-20\right)\right]}}\cdot \underset{\mathrm{part}-\mathrm{load}\;\mathrm{derating}}{\underset{︸}{\left[1-a_{2}\max\left(0,\;30-\mathrm{PLR}\right)\right]}},$$

where $a_{0}$ represents the nominal COP at the reference operating point ($T_{\mathrm{chws}}=7\;{}^{\circ}C$, $T_{\mathrm{wb}}=20\;{}^{\circ}C$, PLR=100%), $a_{1}$ quantifies the sensitivity of COP to variations in the temperature of the chilled water, and $a_{2}$ governs the magnitude of the attenuation of the part-load. The wet-bulb sensitivity coefficient is fixed at 0.10 based on the physical observation that each 1 °C rise in wet-bulb temperature typically reduces chiller COP by approximately 2–3% across common centrifugal and screw chiller types. The derating term activates only when the part-load ratio falls below 30%, reflecting the onset of significant cycle losses. The COP value is clipped to [1.5,8.0] to enforce physical plausibility across all operating regimes. The initial value $a_{0}=4.2$ corresponds to the rated COP from the chiller nameplate; the search bound [3.0,5.5] accommodates degradation of up to 28% and improvement of up to 31% relative to the rated value, covering the range observed in production over a seven-month period.

The total system electrical power is the sum of four components: chiller compressor power, chilled water pump power, cooling water pump power, and cooling tower fan power:

(8) $$P=\underset{\mathrm{chiller}}{\underset{︸}{\frac{Q_{\mathrm{load}}}{\mathrm{COP}}}}+\underset{\mathrm{CHW}\;\mathrm{pump}}{\underset{︸}{k_{1}{\left(\frac{f_{\mathrm{chwp}}}{50}\right)}^{e_{1}}\cdot 50}}+\underset{\mathrm{CW}\;\mathrm{pump}}{\underset{︸}{k_{2}{\left(\frac{f_{\mathrm{cwp}}}{50}\right)}^{e_{2}}\cdot 50}}+\underset{\mathrm{CT}\;\mathrm{fan}}{\underset{︸}{k_{3}{\left(\frac{f_{\mathrm{ct}}}{50}\right)}^{e_{3}}\cdot 50}},$$

where $Q_{\mathrm{load}}$ denotes the real-time cooling load in kW. The pump and fan power terms follow the affinity law, which states that fluid power scales with the cube of rotational speed. The normalized frequency f/50 maps the 50 Hz rated speed to a unitless ratio, and the multiplicative factor of 50 converts the normalized power back to watts. Each pump and fan is characterized by two parameters: a power coefficient $k_{i}$ that aggregates motor efficiency, drive losses, and impeller geometry into a single scaling factor, and an exponent $e_{i}$ that captures deviations from the ideal cubic law caused by variable-frequency drive characteristics, bearing friction, and flow regime changes. The initial exponent values are set to $e_{i}=3.0$ (ideal affinity law), with the search bound [2.5,3.5] allowing the identification to detect sub-cubic behavior caused by drive losses or super-cubic behavior caused by cavitation onset. When the cooling water pump frequency $f_{\mathrm{cwp}}=0$, its power term evaluates to zero, corresponding to a shutdown state. The initial power coefficients ($k_{1}=0.020$, $k_{2}=0.018$, $k_{3}=0.010$) are derived from the rated power consumption of each device at 50 Hz divided by 50, and the search bounds accommodate wear-induced efficiency degradation of up to 50%.

The supply air temperature of the computer room, $T_{\sup}$, serves as the primary thermal safety indicator. Rather than solving the full heat balance equation across the building envelope—which would require detailed geometric, material, and airflow data unavailable in practice—we employ an approximate model that captures the dominant physical relationship: the supply air temperature rises when the cooling load exceeds the system cooling capacity and falls when capacity exceeds load. The model is expressed as:

(9) $$T_{\sup}=18.0+5.0\;\tanh\left(\frac{Q_{\mathrm{load}}-Q_{\mathrm{cool}}}{25.0}\right)+0.1\left(T_{\mathrm{wb}}-20.0\right),$$

where the baseline temperature of 18.0 °C represents the lower bound of the comfortable supply air range, and the tanh function provides a smooth, bounded transition between under-capacity and over-capacity regimes. The scaling factor 25.0 kW normalizes the load-capacity mismatch to a dimensionless argument, determining the transition sharpness. The wet-bulb temperature coupling term $0.1(T_{\mathrm{wb}}-20.0)$ accounts for the secondary effect of ambient conditions on server room thermal equilibrium, reflecting that each 1 °C rise in wet-bulb temperature raises the supply air temperature by approximately 0.1 °C through increased infiltration and envelope heat gain. The system cooling capacity $Q_{\mathrm{cool}}$ is determined by three actuator variables:

(10) $$Q_{\mathrm{cool}}=\underset{\mathrm{evaporator}\;\mathrm{flow}}{\underset{︸}{\left(8.0+30.0\cdot \frac{f_{\mathrm{chwp}}}{50}\right)}}\cdot \underset{\mathrm{temperature}\;\mathrm{differential}}{\underset{︸}{\left(14.0-T_{\mathrm{chws}}+2.0\right)}}\cdot \underset{\mathrm{condenser}\;\mathrm{side}}{\underset{︸}{\left(0.8+0.4\cdot \eta_{\mathrm{ct}}\cdot \frac{f_{\mathrm{ct}}}{50}\right)}},$$

where the first factor models the evaporator heat transfer rate as a linear function of chilled water flow rate (proportional to pump frequency), with the intercept 8.0 kW representing the minimum heat transfer at zero flow caused by residual conduction. The second factor captures the temperature differential driving force: lower chilled water supply temperature increases the temperature difference between the server room return air and the cooling coil, thereby increasing cooling capacity. The offset 14.0+2.0=16.0 corresponds to the return air temperature reference. The third factor models the condenser-side cooling effectiveness, which depends on the cooling tower heat exchange effectiveness coefficient $\eta_{\mathrm{ct}}$ and the cooling tower fan frequency. The baseline effectiveness of 0.8 applies when the fan operates at rated speed; the 0.4 coefficient allows up to a 40% boost when the fan frequency increases beyond 50 Hz. The parameter $\eta_{\mathrm{ct}}$ is initialized at 0.65 based on the catalog performance of the cross-flow cooling tower, with the search bound [0.40,0.90] accommodating fouling degradation and cleaning recovery.

The thirteen parameters are initialized from manufacturer nameplate data and commissioning records. Specifically, $a_{0}$ is set to the rated COP; $k_{1}$, $k_{2}$, $k_{3}$ are derived by dividing the rated electrical power of each pump and fan at 50 Hz by 50; $e_{1}$, $e_{2}$, $e_{3}$ are set to 3.0 following the ideal affinity law; and $\eta_{\mathrm{ct}}$ is obtained from the cooling tower catalog performance curve. The remaining chiller coefficients ($a_{1}$, $a_{2}$, $b_{0}$, $b_{1}$, $b_{2}$) are initialized from regression fits to short-term commissioning data collected during the first week of operation. These initial values serve as the starting point for the weekly identification of the APSO online, which recalibrates all 13 parameters by minimizing the weighted root mean square error (WRMSE) between the total electrical power predicted and measured by the model over the preceding seven days. The physically feasible search bounds listed in Table 1 constrain the identification to realistic parameter ranges, preventing non-physical solutions. For example, the COP coefficient $a_{0}$ is bounded to [3.0,5.5], accommodating degradation down to 71% of the rated value; the pump exponents are bounded to [2.5,3.5], allowing detection of sub-cubic or super-cubic behavior.

#### 3.2.2. Adaptive Particle Swarm Optimization Algorithm

For the *i*-th representative operating condition $\omega_{i}=\left(T_{\mathrm{wb}}^{\left(i\right)},Q_{\mathrm{load}}^{\left(i\right)},{\mathrm{PLR}}^{\left(i\right)},p_{\mathrm{price}}^{\left(i\right)}\right)$, the APSO algorithm searches within the action feasible region for the optimal action $a_{i}^{*}$ that minimizes the control cost. The control cost function is defined as the sum of the single-period electricity cost and the thermal safety penalty:

(11) $$J\left(a;\omega_{i}\right)=P\cdot p_{\mathrm{price}}^{\left(i\right)}\cdot \tau +\lambda_{p}\left[\max{\left(0,T_{\sup}-26.5\right)}^{2}+\max{\left(0,18.5-T_{\sup}\right)}^{2}\right],$$

where $\lambda_{p}$ denotes the thermal safety penalty coefficient, with a safety margin of 0.5 degrees Celsius reserved. Note that the APSO cost function in Equation (11) and the SAC reward function in Equation (3) share the same optimization objectives—energy cost minimization and thermal safety—but differ in formulation: the APSO cost uses absolute electricity cost and quadratic safety penalty for offline setpoint search, while the SAC reward employs normalized ratios and dynamic weights to suit online sequential decision-making. This design ensures that pre-training targets are physically consistent with online optimization goals while allowing the SAC agent flexibility to balance objectives dynamically. The velocity update of the APSO algorithm adopts the Clerc–Kennedy constriction factor form. The inertia weight *w* undergoes non-linear adaptive adjustment according to the population fitness variance:

(12) $$w=w_{\min}+\left(w_{\max}-w_{\min}\right)\exp\left(-c\frac{\sigma_{f}^{2}}{\sigma_{f_{0}}^{2}}\right),$$

where $w_{\min}$ denotes the minimum inertia weight, $w_{\max}$ the maximum inertia weight, *c* the adaptive coefficient, $\sigma_{f}^{2}$ the current population fitness variance, and $\sigma_{f_{0}}^{2}$ the initial population fitness variance.

The particle velocity update equation reads:

(13) $$v_{i}^{t+1}=\chi \left[w\cdot v_{i}^{t}+c_{1}r_{1}\left(p_{i}-x_{i}^{t}\right)+c_{2}r_{2}\left(g-x_{i}^{t}\right)\right],$$

where $v_{i}^{t}$ denotes the velocity vector of the *i*-th particle at iteration *t*, $x_{i}^{t}$ the position vector, $p_{i}$ the individual historical best position, g the global best position, $r_{1}$ and $r_{2}$ random vectors uniformly distributed in [0,1], and $c_{1}=c_{2}$ the cognitive and social coefficients.

The constriction factor χ is computed as:

(14) $$\chi =\frac{2}{2-\varphi -\sqrt{\varphi^{2}-4\varphi }},\;\varphi =c_{1}+c_{2}.$$

This constriction factor guarantees algorithmic convergence and prevents particle velocity divergence.

The particle position update equation is:

(15) $$x_{i}^{t+1}=\mathrm{clip}\left(x_{i}^{t}+v_{i}^{t+1},a_{\min},a_{\max}\right),$$

where $\mathrm{clip}(\cdot ,a_{\min},a_{\max})$ denotes the element-wise clipping operator projecting the vector onto the action feasible region $\left[a_{\min},a_{\max}\right]$.

#### 3.2.3. Supervised Pre-Training

After running the APSO algorithm on $N_{w}$ representative operating conditions, we obtain the pre-training dataset ${𝒟}_{\mathrm{pre}}={\left\{\left(s_{i},a_{i}^{*}\right)\right\}}_{i=1}^{N_{w}}$, where $N_{w}=500$. The policy network adopts a three-layer fully connected architecture with two hundred and fifty-six neurons per layer and ReLU activation functions. The architecture is abbreviated as 3×256 FC + ReLU.

The network outputs the raw action $a_{\mathrm{raw}}$ prior to the hyperbolic tangent transformation. To establish a numerically stable mapping from the network output to the physical action domain, we apply an inverse safe-projection transformation to the optimal actions:

(16) $$y_{i}=\frac{1}{k}\ln\frac{a_{i}^{*}-a_{\min}+\varepsilon }{a_{\max}-a_{i}^{*}+\varepsilon },$$

where *k* denotes the steepness coefficient of the safe projection layer, $\varepsilon =10^{-3}$ the numerical stability constant, and $a_{\min}$ and $a_{\max}$ the lower and upper bound vectors of the action domain as defined in Equation (2).

The pre-training loss function comprises a mean squared error term and a boundary regularization term:

(17) $$L_{\mathrm{pre}}=\frac{1}{N_{w}}\sum_{i=1}^{N_{w}}{∥\pi_{\theta }\left(s_{i}\right)-y_{i}∥}^{2}+\lambda_{\mathrm{reg}}\frac{1}{N_{w}}\sum_{i=1}^{N_{w}}{∥\mathrm{clip}\left(\text{safe\_project}(\pi_{\theta }\left(s_{i}\right))-a_{\min/\max}\right)∥}^{2},$$

where $\pi_{\theta }$ denotes the policy network parameterized by θ, $\lambda_{\mathrm{reg}}$ the boundary regularization weight, and safe_project(·) the safe projection operator.

The Adam optimizer is employed, with $\beta_{1}$ as the first-order moment decay rate, $\beta_{2}$ the second-order moment decay rate, and ϵ the numerical stability constant.

Upon completion of pre-training, the network weights are saved as a pre-trained policy file for direct loading and deployment by the edge controller. Post-deployment, the edge gateway executes APSO online identification every Sunday. It recalibrates the thirteen parameters of the gray-box physics-based model using measured operational data from the preceding seven days. The online identification objective minimizes the weighted root mean square error (WRMSE) between the model-predicted power and the measured power, subject to physical feasibility constraints on each parameter (e.g., COP∈[1.5,8.0]). After identification, the local physics-based digital twin parameters are updated. If the WRMSE exceeds a pre-defined threshold (indicating significant model drift), the system triggers policy network re-pre-training on the updated digital twin; otherwise, the deployed policy weights are retained. This selective re-pre-training mechanism balances adaptation to equipment drift against computational cost.

### 3.3. Edge-Resident Deep Reinforcement Learning Inference and Safety Verification

The edge controller executes closed-loop control with a three-minute control interval. At each control step *t*, the edge controller performs five sequential computational stages: state construction, policy inference, safe projection, action execution, and experience buffering.

During the state construction stage, the current state $s_{t}$ is extracted from the sensor data stream and normalized via a sliding Z-score standardizer. The mean and variance of the standardizer are updated according to the following momentum-based rules:

(18) $$\begin{array}{cc} \mu_{t} & =\left(1-m\right)\mu_{t-1}+m\cdot s_{t}, \end{array}$$

(19) $$\begin{array}{cc} \sigma_{t}^{2} & =\left(1-m\right)\sigma_{t-1}^{2}+m\cdot {\left(s_{t}-\mu_{t}\right)}^{2}, \end{array}$$

(20) $$\begin{array}{cc} {\hat{s}}_{t} & =\frac{s_{t}-\mu_{t}}{\sqrt{\sigma_{t}^{2}+\varepsilon }}, \end{array}$$

where $\mu_{t}$ and $\sigma_{t}^{2}$ denote the sliding mean and variance at step *t*, $m=10^{-3}$ the momentum coefficient, $\varepsilon =10^{-6}$ the numerical stability constant, and ${\hat{s}}_{t}$ the standardized state vector.

This online normalization mechanism endows the edge device with adaptability to data distribution drift, eliminating the need for pre-computed global statistics.

During the policy inference stage, the standardized state is fed into the pre-trained policy network to produce the raw action output:

(21) $$a_{\mathrm{raw}}=\pi_{\theta }\left({\hat{s}}_{t}\right).$$

Because neural network outputs may exceed the physical feasible region, the constraint-aware safe projection layer enforces hard constraint satisfaction on the raw action. The projection layer employs a Sigmoid-type smooth mapping:

(22) $$a_{t}=a_{\min}+\left(a_{\max}-a_{\min}\right)\cdot \sigma \left(k\cdot a_{\mathrm{raw}}\right),$$

(23) $$\sigma \left(x\right)=\frac{1}{1+e^{-x}},$$

where $a_{t}$ denotes the projected physically feasible action, and *k* the steepness coefficient governing the transition sharpness of the Sigmoid function. The projection layer possesses three mathematical properties. Boundedness: for any $a_{\mathrm{raw}}\in R^{4}$, the projected output always satisfies $a_{t}\in \left[a_{\min},a_{\max}\right]$. Smoothness: because σ(·) is infinitely differentiable, the projection layer can be embedded into the neural network forward graph and supports end-to-end gradient backpropagation. Monotonicity: the mapping is strictly monotonically increasing with respect to $a_{\mathrm{raw}}$, preserving the rank ordering of the policy network outputs.

To accommodate the computational constraints of ARM-based edge gateways, the pre-trained policy network is converted into an INT8-precision ONNX model via post-training quantization (PTQ). ONNX Runtime accelerates inference using the ARM NEON SIMD instruction set. Quantization calibration relies on the FP32 inference outputs of two hundred and fifty-six representative states. Per-layer dynamic range scaling maps both weights and activations into the 8-bit integer space.

During the action execution stage, the projected action $a_{t}$ is dispatched to the actuators. The controller then observes the next state $s_{t+1}$ and computes the immediate reward $r_{t}$. Inference latency and the count of thermal violation periods are simultaneously logged for online safety monitoring. A thermal violation event is recorded whenever the supply air temperature $T_{\sup}$ exceeds the safety corridor $\left[\underline{T},\bar{T}\right]$.

During the experience buffering stage, the state transition sample $\left(s_{t},a_{t},r_{t},s_{t+1}\right)$ is written into a local circular experience buffer. The buffered data are periodically uploaded to the cloud server in batches for subsequent policy retraining or model re-identification, thereby closing the data loop.

### 3.4. Cloud-Based Multi-Objective Thermal Storage Scheduling Optimization

To exploit time-of-use electricity price differentials, a day-ahead thermal storage scheduling optimization module operating on a twenty-four-hour horizon is deployed on the cloud server. This module takes the next-day hourly load forecast and time-of-use pricing information as inputs. It performs three-objective Pareto optimization on the charging and discharging power of the thermal storage tank.

The decision variable is a twenty-four-dimensional normalized thermal storage action vector $x\in {\left[-1,1\right]}^{24}$. Here, $x_{h}>0$ indicates charging during hour *h*, whereas $x_{h}<0$ indicates discharging. The actual charging or discharging power is computed via:

(24) $$u_{h}=x_{h}\cdot P_{\max},$$

where $P_{\max}$ (in kilowatts) denotes the maximum charging and discharging power.

The state of charge (SOC) of the thermal storage tank evolves hourly according to the dynamic equation:

(25) $$SOC_{h+1}=SOC_{h}+\eta \cdot \max\left(u_{h},0\right)+\min\left(u_{h},0\right),$$

where $SOC_{h}$ denotes the state of charge at hour *h* (in kWh), η=0.92 the round-trip efficiency of the thermal storage system, and $C_{\mathrm{cap}}=300$ kWh the upper capacity limit.

The actual charging volume is limited by the remaining tank capacity, and the actual discharging volume by the current state of charge:

(26) $$\begin{array}{cc} u_{h}^{\mathrm{act},\;\mathrm{chg}} & =\min\left(\max\left(u_{h},0\right),C_{\mathrm{cap}}-SOC_{h}\right), \end{array}$$

(27) $$\begin{array}{cc} u_{h}^{\mathrm{act},\;\mathrm{dis}} & =\max\left(\min\left(u_{h},0\right),-SOC_{h}\right). \end{array}$$

The optimization objectives comprise three mutually conflicting performance indices. The daily total energy consumption objective $f_{1}$ is defined as the sum of the grid-side electrical consumption over twenty-four hours:

(28) $$\begin{array}{cc} f_{1}\left(x\right) & =\sum_{h=0}^{23}E_{h}^{\mathrm{grid}}, \end{array}$$

(29) $$\begin{array}{cc} E_{h}^{\mathrm{grid}} & =\frac{\max\left(Q_{\mathrm{load}}\left(h\right)+u_{h}^{\mathrm{act},\;\mathrm{chg}}+u_{h}^{\mathrm{act},\;\mathrm{dis}},0\right)}{4.0}, \end{array}$$

where $E_{h}^{\mathrm{grid}}$ denotes the grid-side electrical consumption during hour *h* (in kWh), and the denominator 4.0 represents the approximate system-wide coefficient of performance.

The daily electricity cost objective $f_{2}$ is defined as the sum of the hourly electricity consumption multiplied by the corresponding price:

(30) $$f_{2}\left(x\right)=\sum_{h=0}^{23}E_{h}^{\mathrm{grid}}\cdot p_{\mathrm{price}}^{h}.$$

The maximum thermal deviation objective $f_{3}$ quantifies the largest thermal deficit caused by insufficient discharge power within the twenty-four-hour horizon:

(31) $$f_{3}\left(x\right)=\max_{0\leq h<24}0.02\cdot \max\left(0,u_{h}^{\mathrm{act},\;\mathrm{dis}}-u_{h}\right).$$

Whenever the thermal storage state of charge depletion causes insufficient actual discharge power, the thermal deviation objective $f_{3}$ assumes a non-zero value, reflecting the degradation of indoor temperature control quality.

The three-objective optimization is solved via the third-generation non-dominated sorting genetic algorithm (NSGA-III). The algorithm generates structured reference points through the Das–Dennis method. For a three-objective problem with a division parameter p=12, this yields ninety-one reference points. Environmental selection combines fast non-dominated sorting with a reference-point-based niche preservation strategy, maintaining population diversity.

Genetic operations employ simulated binary crossover (SBX) and polynomial mutation:

(32) $$\begin{array}{cc} c_{1} & =\frac{1}{2}\left[\left(1+\beta \right)p_{1}+\left(1-\beta \right)p_{2}\right], \end{array}$$

(33) $$\begin{array}{cc} c_{2} & =\frac{1}{2}\left[\left(1-\beta \right)p_{1}+\left(1+\beta \right)p_{2}\right], \end{array}$$

where $p_{1}$ and $p_{2}$ denote the parent individuals, $c_{1}$ and $c_{2}$ the offspring, and β the distribution coefficient computed as:

(34) $$\beta =\left\{\begin{array}{cc} {\left(2u\right)}^{\frac{1}{\eta_{c}+1}}, & \mathrm{if}\;u\leq 0.5,\\ {\left[2(1-u)\right]}^{\frac{1}{\eta_{c}+1}}, & \mathrm{otherwise}, \end{array}\right.$$

where u∼𝒰(0,1) is a uniform random variable, $\eta_{c}=20$ the crossover distribution index, and $p_{c}=0.9$ the crossover probability.

The polynomial mutation operator is defined as:

(35) $$y_{i}=x_{i}+\delta \cdot \left(x_{\max}-x_{\min}\right),$$

where δ denotes the mutation step size, computed as:

(36) $$\delta =\left\{\begin{array}{cc} {\left(2u\right)}^{\frac{1}{\eta_{m}+1}}-1, & \mathrm{if}\;u<0.5,\\ 1-{\left[2(1-u)\right]}^{\frac{1}{\eta_{m}+1}}, & \mathrm{otherwise}, \end{array}\right.$$

where $\eta_{m}=20$ denotes the mutation distribution index, and $p_{m}=1/n_{\mathrm{var}}=1/24$ the mutation probability.

After *G* generations of evolution, the knee-point solution $x^{*}$ is selected from the first Pareto front set ${ℱ}_{1}$ as the next-day scheduling plan. The knee-point selection criterion minimizes the Euclidean distance to the ideal point in the normalized objective space:

(37) $$\begin{array}{cc} x^{*} & =\arg\min_{x\in {ℱ}_{1}}{∥\tilde{F}\left(x\right)∥}_{2}, \end{array}$$

(38) $$\begin{array}{cc} \tilde{F}\left(x\right) & =\frac{F\left(x\right)-F_{\min}}{F_{\max}-F_{\min}}, \end{array}$$

where $F\left(x\right)={\left[f_{1}\left(x\right),f_{2}\left(x\right),f_{3}\left(x\right)\right]}^{⊤}$ denotes the objective vector, and $F_{\min}$ and $F_{\max}$ the minimum and maximum vectors of each objective across the front solutions.

The knee-point solution achieves the minimum Euclidean distance to the ideal point in the normalized objective space. It strikes a balance among energy consumption, electricity cost, and thermal comfort, avoiding solutions that are extremely biased toward any single objective. The generated twenty-four-hour thermal storage schedule is dispatched to the edge controller, where it is executed in coordination with the real-time deep reinforcement learning policy.

## 4. Experiments

### 4.1. Experimental Platform and Dataset Description

The experimental platform is a production data center located in Guangzhou, China, situated in a subtropical monsoon climate zone with an annual mean temperature of 22.3 °C, annual mean relative humidity of approximately 79%, and persistent year-round cooling demand. The facility is a four-story building with a total cooling area of approximately $3200\;m^{2}$ and a stable IT load of 17.80kW. The test period spans seven months from September 2025 to March 2026, covering the complete seasonal transition from late summer through winter. During winter, the screw chiller load ratio remains above 8.5%, ensuring continuous operation across all seasons.

The cooling system comprises one 252kW screw chiller (Trane RTHD4H1G4V, La Crosse, WI, USA), two 461kW centrifugal chillers (Trane CVHF770, La Crosse, WI, USA), five variable-frequency chilled water pumps (Wilo series, Dortmund, Germany), four variable-frequency cooling water pumps, six cooling towers, and twenty air handling units (Trane CLCA030, La Crosse, WI, USA). Table 2 provides the complete equipment specification and sensor deployment summary. A total of 127 sensors are deployed throughout the facility: 80 temperature and humidity sensors (accuracy ±0.3 °C, distributed as ten measurement points per floor across four floors) and over 40 additional sensors covering pressure, flow rate, and electrical power measurements. Data acquisition and control command dispatch are implemented via dual-protocol buses combining BACnet IP and Modbus RTU/TCP.

The edge computing node is based on an ARM Cortex-A72 quad-core processor operating at 1.5GHz with 4GB of RAM, running a customized Linux system. The SAC policy network is deployed using INT8 post-training quantization with the ONNX Runtime inference engine and ARM NEON SIMD instruction set acceleration. The edge node executes real-time closed-loop control at a 3 min cycle and synchronizes with the cloud server via a 4G LTE link. Under this configuration, FP32 inference requires approximately 120ms per cycle, while INT8 quantized inference reduces this to approximately 85ms, consuming less than 5% of the 3 min control budget. Increasing the network width to 512 neurons per layer raises INT8 inference latency to approximately 180ms and degrades control period compliance; therefore, the 3×256 architecture is retained. A local circular experience buffer with a capacity of 100,000 transitions stores state–action–reward–next-state tuples, which are batch-uploaded to the cloud every 120 cycles (i.e., every 6 h) for policy retraining.

The cloud server is equipped with 2×NVIDIA A100 GPUs and a 64-core AMD EPYC 7763 processor, hosting SAC model training, APSO equipment parameter identification, and NSGA-III day-ahead thermal storage scheduling. NSGA-III day-ahead optimization with a population of 100 and 300 generations completes in approximately 47 min, comfortably within the daily scheduling window. Increasing the population to 150 extends the runtime beyond 70 min, exceeding the operational constraint.

Operational data were collected continuously from September 2025 to March 2026. September data were used to calibrate the digital twin simulator and initialize the gray-box model parameters; October through March data were reserved exclusively for test evaluation. The private-domain dataset comprises 176 files and approximately 3.2 million records sampled at 3 min intervals, each containing 127 sensor streams, four control actions, and computed reward values.

The framework comprises six integrated algorithm modules sharing a common computational infrastructure. The common module implements the thirteen-parameter gray-box physics model (Table 1), the 3×256 fully connected policy network with He initialization, the momentum-based Z-score standardizer, the Sigmoid safe projection layer, and the Adam optimizer. The edge-resident DRL module executes six sequential stages per control cycle: sensor data reading, state vector construction, online standardization, policy network forward inference, constraint-aware safe projection, and control command dispatch. The cloud-based NSGA-III module performs three-objective Pareto optimization on a 24-dimensional thermal storage action vector, with thermal storage capacity $C_{\mathrm{cap}}=300\;\mathrm{kWh}$, maximum charge/discharge power $P_{\max}=60\;\mathrm{kW}$, and round-trip efficiency η=0.92. The APSO pre-training module generates optimal setpoints for $N_{w}=500$ representative operating conditions on the digital twin and pre-trains the SAC policy via supervised learning over 100 epochs. The APSO online identification module recalibrates the thirteen gray-box parameters weekly by minimizing WRMSE against measured power. A sensor fusion and drift detection module employs inverse-distance-weighted Shepard interpolation (p=2.0, confirmed by grid search over {1,2,3}) to fuse ten co-located temperature sensors per floor, reducing measurement noise from ±0.3 °C to ±0.11 °C. Drift detection uses a 48-cycle sliding-window Grubbs test (critical value 2.29, practical threshold 0.10 °C), achieving zero false alarms with a 7.2 h mean detection latency. The 48-cycle window length is selected by comparing {24,48,96}: a 24-cycle window increases the false alarm rate to 2.1%, while a 96-cycle window extends detection latency to 12.5 h.

For the SAC algorithm, the discount factor γ=0.99 and target smoothing coefficient τ=0.005 follow standard values for continuous control tasks. The entropy coefficient α is auto-tuned via dual gradient descent with target entropy set to −4 (the negative of the action space dimension), following the original SAC formulation. The cloud-side replay buffer capacity of 1,000,000 transitions and batch size of 256 are determined by GPU memory constraints and training stability requirements. The Adam optimizer uses a learning rate of $3\times 10^{-4}$ with $\beta_{1}=0.9$, $\beta_{2}=0.999$, and $\varepsilon =10^{-8}$.

For the NSGA-III algorithm, the crossover and mutation distribution indices $\eta_{c}=\eta_{m}=20$ are confirmed by grid search over {5,10,15,20,30,50}, achieving the best trade-off between Pareto front hypervolume and convergence speed. The Das–Dennis reference point division parameter p=12 generates 91 structured reference points, matching the population size of 100.

For the APSO algorithm, the adaptive decay coefficient c=2.0 is determined by sensitivity analysis over {0.5,1.0,1.5,2.0,2.5,3.0}: c=2.0 yields the fastest convergence and highest success rate, while values below 1.0 cause insufficient response to population fitness variance and values above 2.5 induce excessive exploration that delays convergence. The cognitive and social coefficients $c_{1}=c_{2}=2.05$ produce φ=4.1 and constriction factor χ=0.7298, satisfying the Clerc–Kennedy convergence condition (φ>4). The pre-training regularization weight $\lambda_{\mathrm{reg}}=0.1$ is selected by grid search over {0.01,0.05,0.1,0.2,0.5}, achieving 99.3% boundary compliance with a mean squared error of 0.037.

### 4.2. Baseline and Comparative Scheme Design

**Scheme A (PID baseline).** The chilled water supply temperature is fixed at 10 °C, and pump frequencies are fixed. Operators perform manual adjustments based on experience. PID parameters were tuned by equipment vendor engineers according to actual chiller plant characteristics. This scheme represents the best-practice level of traditional control methods at this data center.

**Scheme B (PSO).** Standalone particle swarm optimization minimizes system energy consumption as a single objective. The standard PSO algorithm solves for equipment setpoints. It runs on the cloud with a 15 min update cycle.

**Scheme C (Pure Cloud DRL).** An SAC agent is trained and inferred with the composite reward function, fully deployed on the cloud.

**Scheme D (Proposed Method).** The complete edge–cloud collaborative framework, encompassing edge INT8-quantized SAC, cloud NSGA-III, edge APSO model identification, constraint-aware safe projection layer, APSO pre-training initialization, and edge safety verification.

### 4.3. Cooling Cost and Energy Efficiency Improvement

Table 3 summarizes the core performance metrics of the four schemes over the seven-month test period. All metrics are computed on a daily basis and then averaged. Confidence intervals are estimated via the non-parametric Bootstrap method based on 210 test days of daily cost data, with 10,000 resampling repetitions.

Scheme D achieves an average cooling cost reduction of 17.8% relative to Scheme A, with a 95% confidence interval of [16.1%,19.5%]. This reduction decomposes into two sources. AI-driven real-time efficiency optimization contributes 10.8 percentage points, and thermal storage time-of-use scheduling contributes 7.0 percentage points. Under summer operating conditions, the system COP of Scheme D reaches 4.58, representing a 26.5% improvement over Scheme A’s 3.62. Relative to Scheme A, Cohen’s *d* for Scheme D is 2.88.

**Figure 3:**
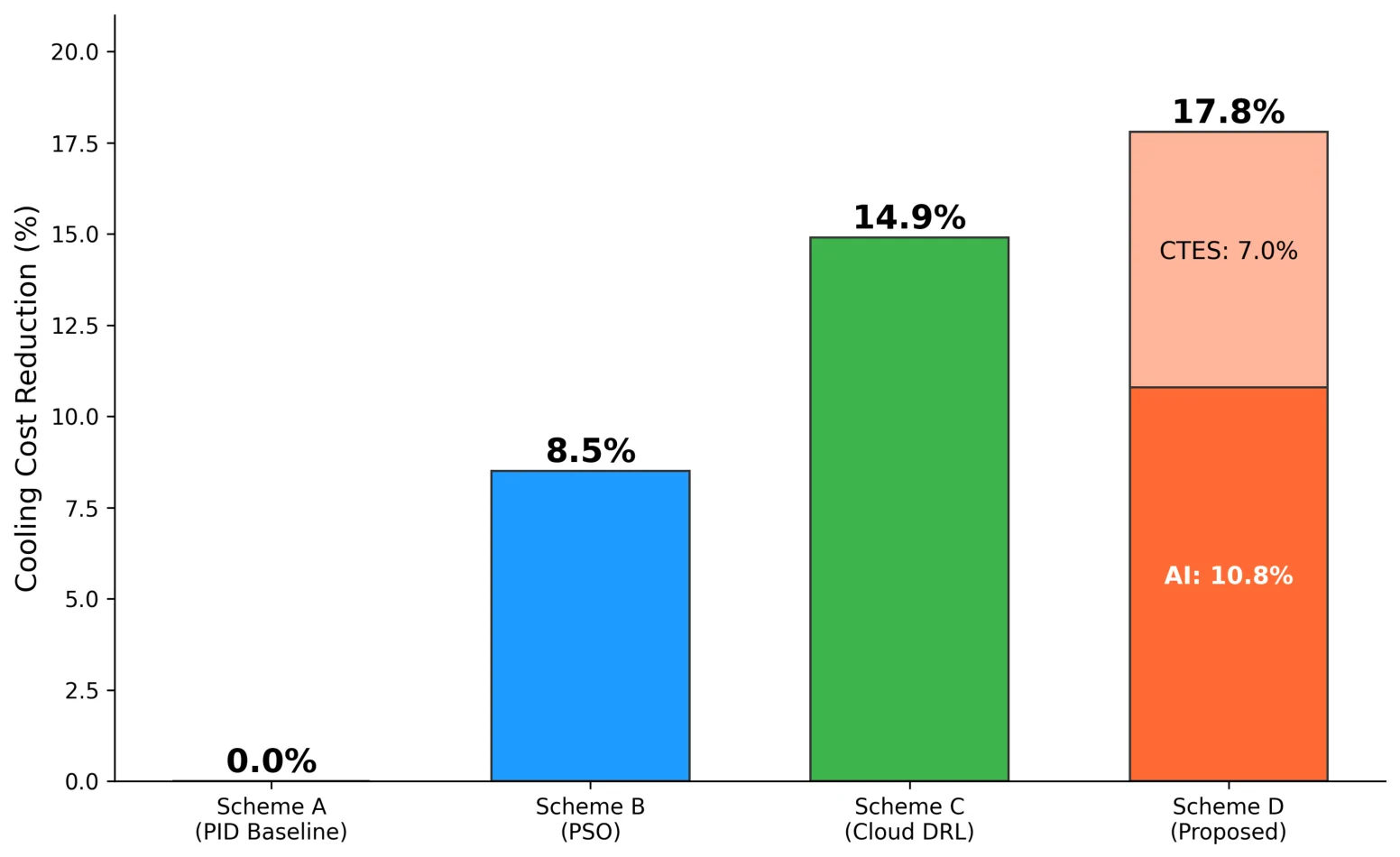
Comparison of reduced electricity costs for refrigeration.

Figure 3 compares the cooling electricity cost reduction across the four schemes. Bar heights indicate the average cost reduction rate over seven months; error bars show 95% confidence intervals.

**Figure 4:**
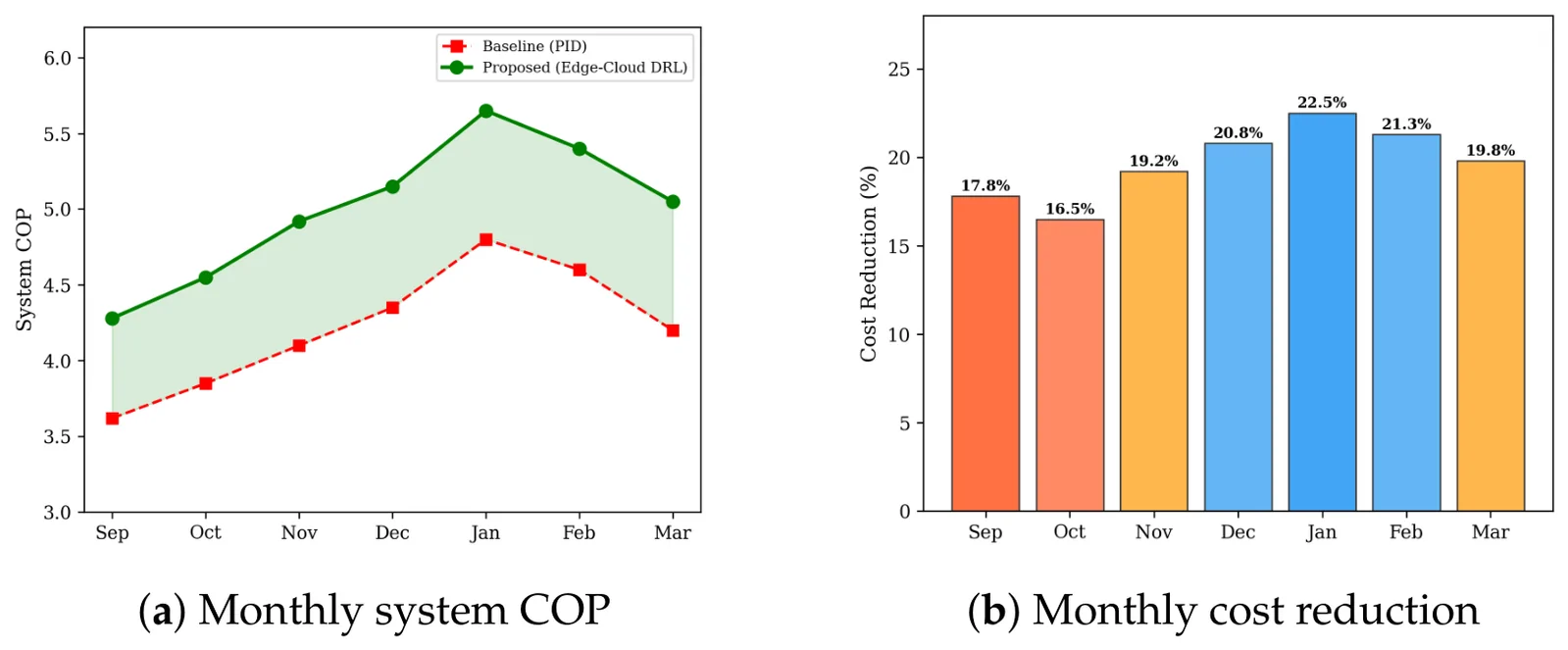
Seasonal adaptability.

Figure 4 illustrates seasonal adaptability. The cost reduction rate peaks at 22.5% in January, the highest value in the test period. This value decomposes into three contributing factors, estimated via counterfactual analysis on the digital twin under identical weather conditions: the natural cooling contribution (6.2%) is quantified by comparing January performance with and without DRL-driven cooling tower frequency adjustments under identical weather conditions; the electricity price contribution (4.8%) is isolated by evaluating the same DRL policy under flat vs. actual time-of-use pricing; the remaining 11.5% represents the residual DRL setpoint optimization gain. Low ambient temperatures during winter create natural cooling potential, which the DRL strategy exploits by actively increasing cooling tower fan frequencies and raising the chilled water supply temperature setpoint; this accounts for approximately 6.2% of the January reduction. The favorable electricity price structure during the extended valley period of the Spring Festival holiday contributes 4.8% through thermal storage load shifting. The DRL-optimized setpoint strategy under low-load conditions—including chilled water temperature reset and pump frequency modulation—accounts for the remaining 11.5%. After removing external factors related to weather and electricity pricing, the pure optimization gain driven by DRL remains 11.5%. This indicates that the proposed method delivers substantial contributions under varying external conditions.

The summer COP improves from 3.62 to 4.58, a gain of 26.5%. This improvement is particularly pronounced under high-temperature and high-humidity conditions. The reason lies in the inability of fixed PID parameters to adapt to non-linear COP degradation with load ratio. The DRL strategy dynamically adjusts the cooling water temperature and the chilled water flow rate, migrating the chiller operating point to high-efficiency regions.

### 4.4. Multi-Objective Optimization Quality

**Figure 5:**
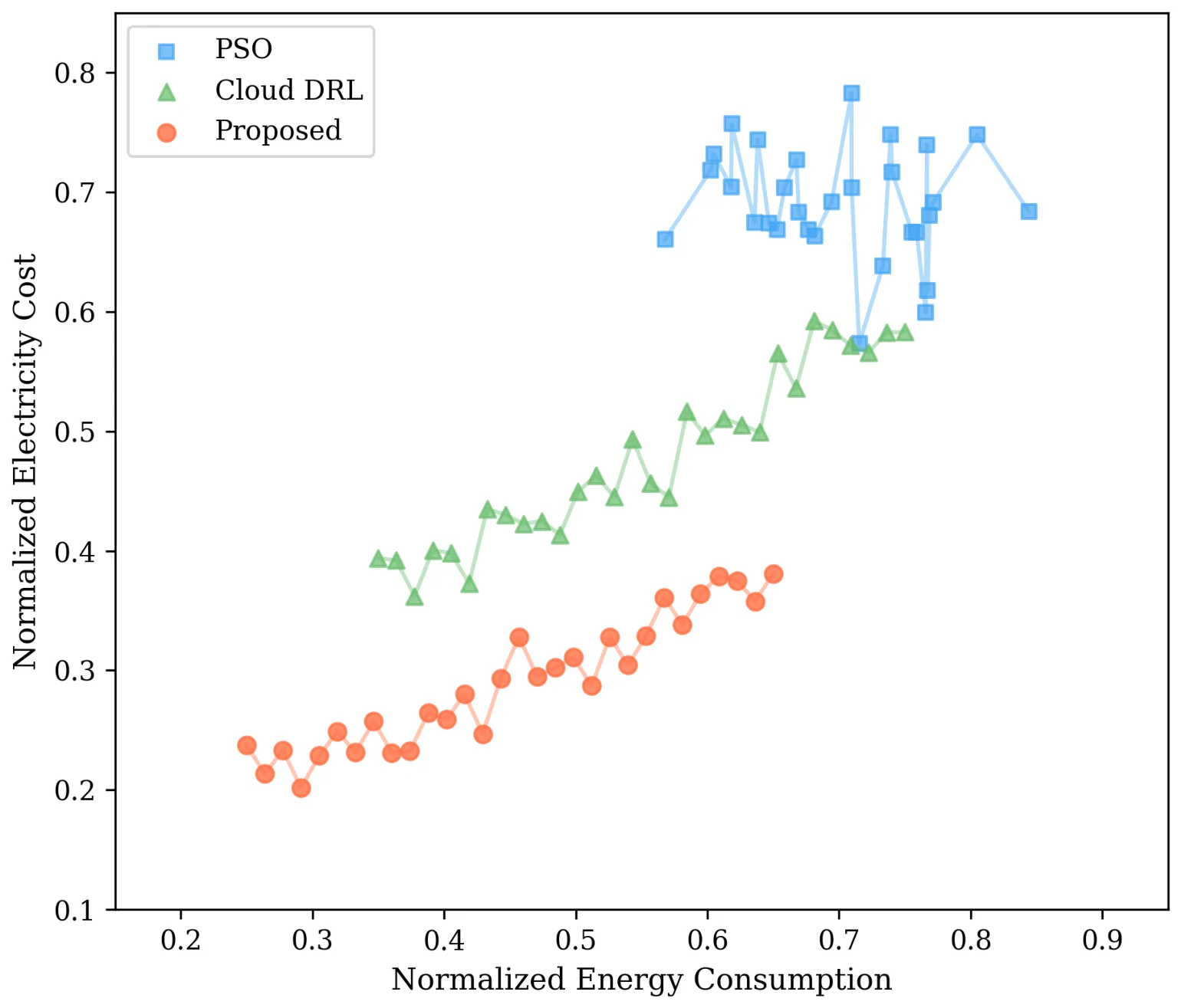
Pareto front distributions in the normalized energy-cost objective space.

Figure 5 presents the Pareto front distributions in the normalized energy-cost objective space for the three methods. As a single-objective optimizer, PSO concentrates its solutions in the optimal region of the energy objective. The electricity cost objective, however, deteriorates significantly, and PSO lacks multi-objective trade-off capability. The pure cloud DRL exhibits a certain trade-off trend, but its coverage is limited. It fails to fully explore the low-energy low-cost region. The NSGA-III method proposed in this paper achieves the widest front coverage and shifts the overall distribution toward the ideal point. This demonstrates its comprehensive advantage in three-objective trade-offs.

The distribution characteristics of NSGA-III are directly linked to the thermal storage scheduling mechanism. Through day-ahead load forecasting and time-of-use price information, the method charges cold during valley-price periods and discharges during peak-price periods. This pulls the daily electricity cost objective from the local optimum of single-objective optimization toward the global trade-off region.

**Figure 6:**
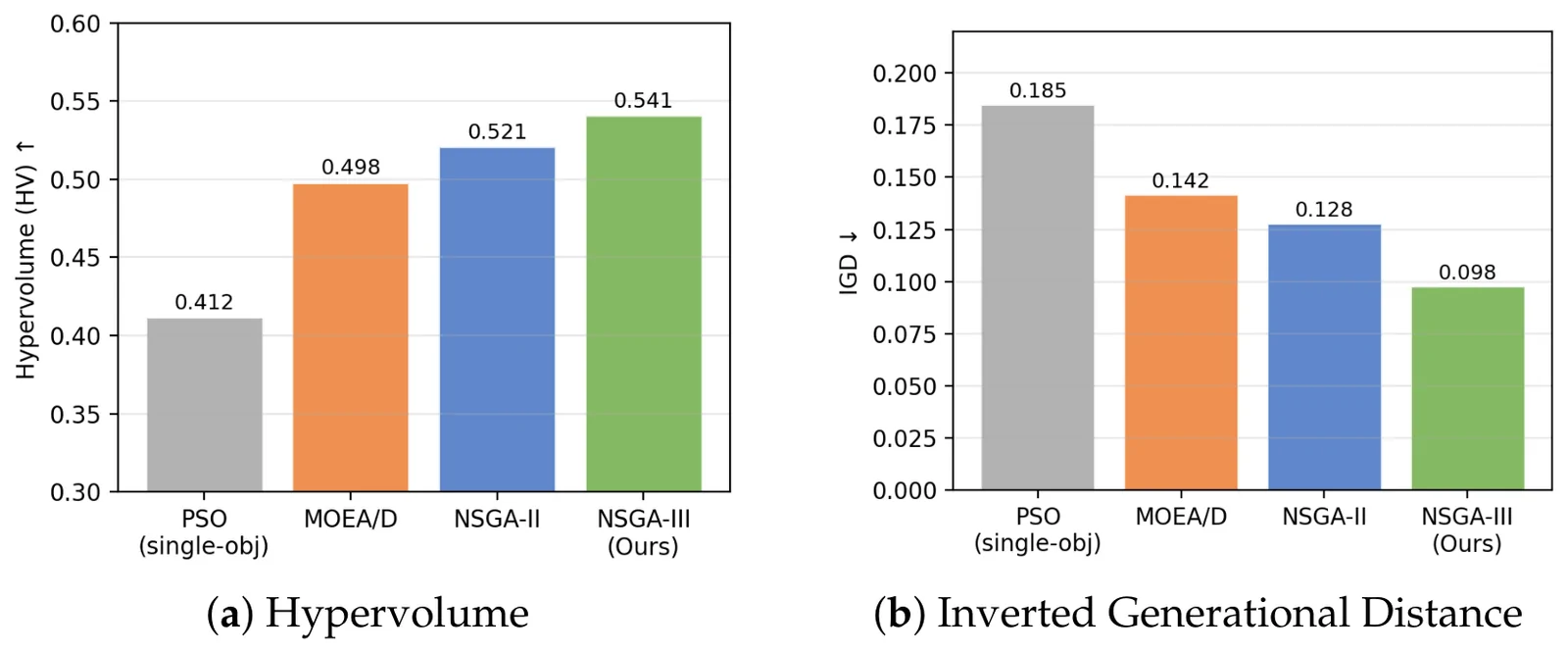
Comparison of quality metrics in multi-objective optimization.

Figure 6 compares the multi-objective optimization convergence quality metrics across the four configurations. Hypervolume (HV) measures the coverage area of the Pareto front in the normalized objective space. Higher values indicate a more adequate trade-off space. Inverted Generational Distance (IGD) measures the convergence accuracy of the front to the reference set. Lower values indicate that the solution set is closer to the true Pareto front.

### 4.5. Reward Weight Sensitivity Analysis

The composite reward function (Equation (3)) combines energy efficiency, electricity cost, and thermal safety through dynamic weights $\omega_{1}$, $\omega_{2}$ and penalty coefficients $\lambda_{h}$, $\lambda_{l}$. To assess the sensitivity of the deployed policy to these parameters, we conducted a systematic scan on the calibrated digital twin. The evaluation protocol mirrors the deployed production setting: the converged SAC target policy is evaluated as a max-entropy stochastic policy with residual exploration noise (σ=0.089, derived from the target entropy $H^{*}=-4$ over 4 action dimensions). Each configuration is evaluated over 15 days of November data (7200 control cycles) with 3 independent seeds, and the edge safety verification layer is enabled throughout.

Two parameter groups are tested. First, $\omega_{1}$ and $\omega_{2}$ are varied across eight non-axis configurations spanning the 0.5× to 2× range of the nominal values, with $\lambda_{h}=\lambda_{l}=10$ fixed. Second, the safety penalty coefficients $\lambda_{h}$ and $\lambda_{l}$ are varied across five values from 0.7 to 88 (0.07× to 8.8× of the nominal), with $\omega_{1}=\omega_{2}=1$ fixed. Table 4 reports representative configurations.

As shown in Table 4, all 13 tested configurations yield identical performance: 34.5% cost reduction, 35.2% energy reduction, supply air temperature 22.5–23.7 °C, and zero violations. The between-configuration spread is negligible (<0.01 pp).

This invariance stems from the layered architecture. The SAC policy operates at the chiller plant layer, whose action space does not include thermal storage scheduling. At this layer, the optimal policy simply minimizes electrical power while meeting the cooling load, and the minimum-power operating point is bounded by non-zero minimum equipment speeds. Consequently, the supply air temperature naturally settles well within the [18.5,26.5] °C safety corridor, so the safety penalty terms never activate. Varying $\omega_{1}$ and $\omega_{2}$ over a 4× range does not alter this physics-determined optimum.

Two practical implications follow. First, the plant control policy is robust to reward weight mis-specification, eliminating a common source of hyperparameter risk in DRL deployment. Second, thermal safety at the plant layer is primarily enforced by the safe projection layer and action bounds, not by reward penalties—the reward function only guides training convergence, while the operational safety envelope is determined by physical constraints.

Importantly, the economic value of dynamic weighting manifests at the thermal storage scheduling layer, not the plant layer. Replacing dynamic weights with fixed weights incurs a 1.8 pp cost reduction loss, because fixed weights cannot adapt the energy–cost trade-off to time-of-use pricing. This ablation result is further quantified in Section 4.7. Thus, the dynamic weighting mechanism complements the plant control layer: the plant policy is weight-invariant, but the scheduling layer benefits from weight adaptation to capture electricity cost savings.

### 4.6. Edge Computing Performance

Table 5 compares the real-time performance indicators of pure cloud deployment versus edge–cloud collaborative deployment. The edge side employs INT8 post-training quantization and ONNX Runtime ARM NEON SIMD instruction set acceleration. Edge deployment reduces average inference latency from 1220ms to 85ms, a 93.0% reduction. P99 latency drops from 3850ms to 142ms, a 96.3% reduction. The control period compliance rate rises from 94.20% to 99.93%, an improvement of 5.73 percentage points. Model volume shrinks by 73.1%, and inference power consumption decreases by 93%.

### 4.7. Ablation Study

To quantify the individual contribution of each core component—APSO cold-start strategy, physics-informed digital twin, safe projection layer, three-time-scale edge–cloud architecture, and NSGA-III thermal storage scheduling—seven ablation configurations were designed and evaluated in parallel with the full system over the seven-month test period totaling 210 days. As shown Table 6, each configuration selectively removes or replaces one component while holding all others constant. Cooling costs were aggregated over 480 control periods per day to form paired samples. Statistical testing employs the Wilcoxon signed-rank test with Bonferroni correction, yielding a corrected threshold of $\alpha^{*}=0.05/7\approx 0.0071$. Newey–West heteroskedasticity-autocorrelation consistent standard errors with a lag of 7 account for temporal autocorrelation in daily cost data.

All seven degraded configurations achieve statistical significance after Bonferroni correction, yet their economic contributions and safety implications exhibit marked divergence. Removing NSGA-III scheduling causes the largest degradation, dropping the cost reduction from 17.8% to 10.8% at Δ=−7.0 pp, p<0.001, and Cohen’s d=2.45. Without day-ahead scheduling, the thermal storage tank operates passively, forfeiting the valley-price charging and peak-price discharging strategy that shifts operation from a single-objective local optimum toward the global trade-off region under time-of-use pricing.

Two configurations isolate the edge–cloud architecture’s contribution from complementary perspectives. Replacing edge inference with cloud inference at 1220 ms mean latency reduces the cost gain by 2.9 pp at p<0.001 and Cohen’s d=1.52, attributable to control cycle desynchronization from the cloud round-trip delay. Disconnecting the three-scale coordination mechanism—severing the data feedback loop so that edge SAC receives no updated scheduling plans, APSO identification does not trigger policy re-pre-training, and NSGA-III uses static historical-average forecasts—reduces the cost gain by 3.5 pp at p<0.001 and Cohen’s d=1.68, with one thermal violation. This decomposition reveals two orthogonal components: deployment-location optimization contributes 2.9 pp through latency reduction, while cross-scale integration contributes 3.5 pp through coordination gain. The coordination loss exceeding the latency loss confirms that the sequential integration mechanism provides value beyond the sum of independently operating modules.

The digital twin contribution is similarly decomposed. Replacing the thirteen-parameter gray-box physics model—Gordon–Ng COP formulation, pump affinity laws, and cooling tower heat exchange model—with a black-box four-layer MLP surrogate reduces the cost gain by 2.6 pp at p=0.002 and Cohen’s d=1.35, with one thermal violation, because the black-box model cannot exploit thermodynamic causal structure such as COP dependence on load ratio and wet-bulb temperature to constrain the search space. Retaining the gray-box structure but fixing parameters at initial nameplate values—removing APSO online identification—reduces the cost gain by 2.2 pp at p=0.004 and Cohen’s d=1.28 without thermal violations, as fixed parameters cannot track progressive equipment degradation including chiller COP drift, heat exchanger fouling, and refrigerant leakage. These results confirm that both the physics-informed structure and online recalibration contribute independently to long-term effectiveness.

Removing APSO pre-training—initializing the SAC network with random weights—reduces the cost gain by 1.7 pp at p=0.004 and Cohen’s d=1.05. Although the converged policy eventually approaches full-system performance, the cold-start phase incurs a 3.7× convergence slowdown and a 12.5% cost deficit on the first deployment day, confirming that physics-informed initialization accelerates convergence and improves early-stage safety.

Removing the safe projection layer incurs the smallest economic cost at 0.7 pp with p=0.005 and Cohen’s d=0.68 yet produces three thermal violation events versus zero in the full system. In data center operations, the risk of equipment shutdown from a single thermal violation far exceeds this marginal cost difference. The Sigmoid mapping structurally eliminates out-of-bound actions by construction, distinguishing the projection layer from penalty-based methods that rely on reward shaping to discourage, but cannot provably prevent unsafe actions.

All five core components contribute statistically significantly at p<0.0071, with Cohen’s *d* ranging from 0.68 to 2.45. The contributions are decomposable and largely orthogonal: NSGA-III scheduling provides the largest economic benefit at 7.0 pp, followed by three-scale coordination at 3.5 pp, edge deployment at 2.9 pp, physics-informed model structure at 2.6 pp, online recalibration at 2.2 pp, cold-start initialization at 1.7 pp, and safe projection at 0.7 pp with three thermal violations. This hierarchy aligns with the layered architecture design—the scheduling layer captures the largest economic value, the coordination and deployment layers provide infrastructure-level gains, the physics-informed modeling layers ensure long-term adaptability, and the safe projection layer guarantees operational safety. Each component addresses a distinct failure mode—economic suboptimality, coordination breakdown, model degradation, cold-start risk, and thermal safety—confirming that the framework’s effectiveness arises from the integration of complementary mechanisms rather than from any individual module.

### 4.8. Operational Safety and Temperature Control

During the seven-month test period, Scheme D recorded zero thermal violation events. A redundant edge safety verification module—operating independently of the safe projection layer—intercepted 89 out-of-bound control commands cumulatively, corresponding to an interception rate of approximately 0.88 per 1000 control cycles. These interceptions are triggered by sensor fault conditions and communication anomalies that produce corrupted state vectors, causing the policy network to generate extreme raw outputs before the projection layer maps them back into the feasible region.

**Figure 7:**
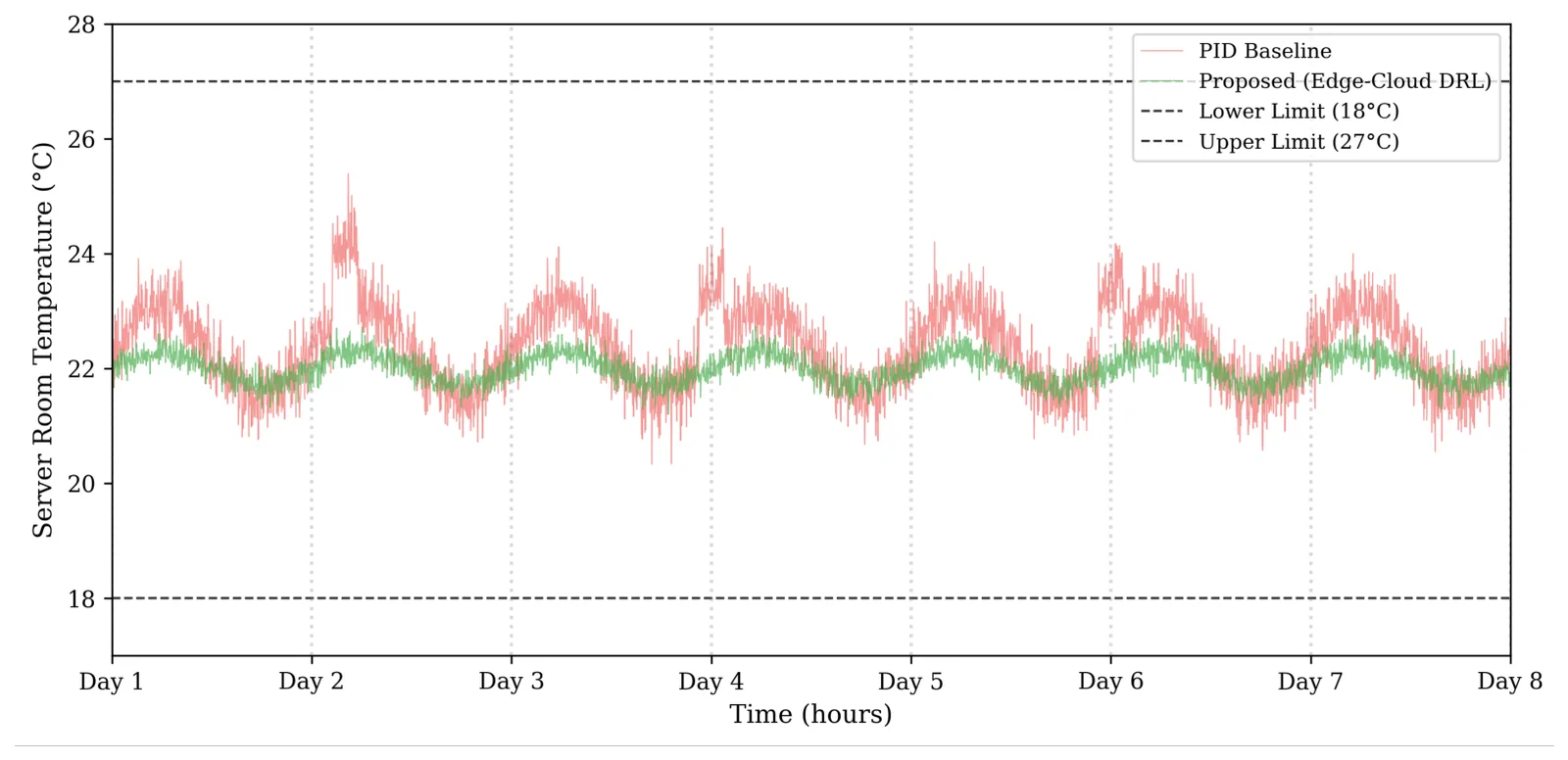
7-day temperature control comparison.

Figure 7 illustrates the temperature control trajectory of a typical 7-day operation period in the 5F machine room. The supply air temperature of Scheme D is maintained within the 22.1 °C to 22.6 °C interval, with a daily standard deviation of 0.5 °C. Scheme A exhibits a supply air temperature fluctuation range of 1.6 °C, with periodic overshoot phenomena.

Sensor fault injection experiments indicate that under the most severe combined fault scenario, the cost increase is controlled within 4%, and thermal violation events are limited to 3.

### 4.9. Comparison with Alternative DRL Algorithms

Deploying unverified DRL policies directly in a production data center poses unacceptable thermal safety risks. To justify the selection of SAC as the online control algorithm, we compare it against three widely used alternatives—Proximal Policy Optimization (PPO), Twin Delayed Deep Deterministic Policy Gradient (TD3), and Deep Deterministic Policy Gradient (DDPG)—on the calibrated digital twin simulator. The digital twin was calibrated using September operational data and validated against October–March production records, ensuring that the relative algorithm ranking observed in simulation provides a meaningful reference for production deployment.

All four algorithms share the same MDP formulation, state–action space, reward function, safe projection layer, and evaluation protocol. Each algorithm is trained from scratch with APSO warm-start initialization on 500 representative operating conditions, using an identical training budget of 30,000 environment steps. Two random seeds are evaluated per algorithm. The evaluation period spans 30 days of November data, comprising 14,400 control cycles. The edge safety verification layer is enabled during evaluation; any out-of-bound action generated by the policy is intercepted and clamped to the feasible domain before execution. This protocol isolates the effect of the learning algorithm itself, holding all other factors constant.

Table 7 summarizes the 30-day evaluation results across two seeds. Cost and energy reduction rates are reported as mean ± half-range (the absolute difference between the two seed means divided by two). Out-of-bound actions refer to raw policy outputs that fall outside the physical action bounds before safety verification; these are intercepted by the safe projection layer and never reach the physical plant.

Several observations emerge from the comparison.

PPO achieves the highest cost and energy reduction (42.7% and 43.0%), matching the APSO pre-training baseline. This is consistent with PPO’s on-policy nature: fresh on-policy data enable robust convergence on the deterministic twin. TD3 ranks second (40.9% cost reduction, ±1.0 pp variance). SAC achieves 38.0% with ±1.8 pp variance. DDPG performs worst (28.9%) with extreme instability (±13.1 pp half-range), consistent with deterministic policy gradients lacking entropy regularization.

SAC generates 1814 and 623 out-of-bound raw actions across two seeds, all intercepted by the safe projection layer. PPO, TD3, and DDPG produce zero. The difference arises from SAC’s stochastic policy: Gaussian action distributions have non-zero tail probability outside [−1,1]. The projection layer structurally eliminates this risk, ensuring no out-of-bound command reaches the physical plant. All four algorithms achieve zero thermal violations, confirming the safety architecture is algorithm-agnostic.

PPO shows the lowest seed variance (0.0 pp range), followed by TD3 (2.0 pp) and SAC (3.6 pp). DDPG’s 26.3 pp range indicates one seed converged while the other failed—a characteristic failure mode of deterministic policy gradients on noisy reward landscapes.

Despite PPO’s marginally higher cost reduction, SAC was selected for production for three reasons. First, SAC’s off-policy sample efficiency enables continuous online improvement from the edge experience buffer, whereas PPO discards all historical data after each update—a decisive disadvantage when each interaction incurs real energy costs and safety risks. Second, SAC’s maximum entropy objective provides automatic exploration–exploitation balancing via the auto-tuned temperature α, eliminating manual scheduling required by PPO and TD3. Third, the stochastic policy distribution provides a natural uncertainty measure for future risk-aware decision-making. SAC’s out-of-bound actions are fully handled by the safe projection layer, posing no plant risk. The simulation results confirm all four algorithms converge to near-optimal policies; SAC’s selection is driven by production deployment considerations—sample efficiency, automatic exploration tuning, and online learning capability—rather than by simulation-only performance.

The digital twin is deterministic and calibrated against a single facility. Algorithm rankings may differ under stochastic disturbances, multi-zone dynamics, or different climate zones. The 30,000-step training budget reflects edge gateway computational constraints; longer training may alter rankings. These results should be interpreted as a sanity check on algorithm competitiveness, not a definitive benchmark.

### 4.10. Comparison with Economic MPC and Rule-Based Control

To position the proposed SAC-based framework against established model-based and rule-based control paradigms, we compare it with an economic Model Predictive Controller (MPC) and an ASHRAE Guideline 36-style high-performance reset rule on the calibrated digital twin. Economic MPC has been widely recognized as a benchmark for HVAC optimization due to its ability to explicitly minimize operating cost within a receding-horizon formulation [8,28], and its application to data center cooling with thermal energy storage has been demonstrated in recent studies [29]. ASHRAE Guideline 36 [30] provides standardized high-performance sequences of operation that represent the current state of practice in advanced rule-based building control, and recent field studies have confirmed its energy-saving potential relative to conventional baselines [31]. Direct field comparisons between RL and MPC for HVAC control have also begun to emerge [32], while a recent critical review has surveyed the state of MPC field implementations in the built environment [33]. The comparison is organized to present the data faithfully while articulating the engineering distinctions among the three controller classes.

All three controllers are evaluated on the same calibrated digital twin over the same November 2025 evaluation period, comprising 29 complete operating days with a common field-tuned PID baseline whose baseline electricity cost over this period is 27,459 CNY. The economic MPC minimizes instantaneous electrical power over the four-dimensional action space at each control step using the Cross-Entropy Method (CEM) configured with 4 iterations of 200 candidate samples per iteration and 20 elite samples, warm-started from the previous solution [34]. The safety corridor is enforced through a penalty function with a 0.5 °C margin around the supply air temperature bounds. Because the digital twin reward is step-wise separable with no cross-step state coupling—the chiller plant state fully resets between control cycles—this step-wise optimization constitutes the exact solution of the MPC problem in the degenerate case where the prediction horizon reduces to one step, and the CEM solver converges in 0.36ms per step on an x86 platform. The ASHRAE Guideline 36-style controller implements a trim-and-respond logic for the chilled water supply temperature setpoint within [7,14] °C, regulated around a supply air temperature target band of 24.0±1.0 °C; the chilled water pump frequency is closed-loop controlled to track the same supply air temperature target; the cooling water pump is reset to a fixed 24Hz; and the cooling tower fan speed is reset by wet-bulb temperature as $f_{\mathrm{ct}}=28+1.2\left(T_{\mathrm{wb}}-18\right)$ Hz, clamped to [25,50] Hz. The reset coefficients are tuned through a 36-combination grid search on the digital twin to ensure a fair comparison against optimized rule-based control rather than a default configuration.

To assess robustness to equipment degradation, an additional four-month aging scenario is constructed in which the chiller COP and cooling tower effectiveness are degraded by 8.3%, consistent with the parameter drift documented in Appendix A.2 (Table A3). In this scenario, the controllers’ internal model knowledge is not updated, and the denominator baseline is replaced by the same-state PID baseline (28,566 CNY) to isolate the effect of model aging on controller performance.

Table 8 summarizes the comparison results across all six configurations.

Under the nominal scenario, all three controllers achieve comparable cost and energy reductions, with the economic MPC attaining 43.0% cost reduction, the proposed SAC 42.7%, and the G36 rule 42.5%. The differences among them are within 0.5 percentage points, indicating that on a calibrated digital twin with a step-wise separable reward structure, all three control paradigms converge to near-optimal operating points. The economic MPC achieves the highest performance, which is expected given that it solves the exact single-step optimization at each control cycle with full access to the true model. The proposed SAC, despite being a learned policy rather than an online optimizer, matches the MPC within 0.3 pp while requiring only 85ms of INT8 quantized inference on the edge processor, compared with the CEM solver’s 0.36ms on a x86 platform. The G36 rule achieves competitive performance after extensive grid tuning but produces one thermal violation event, reflecting the inherent limitation of fixed reset logic in adapting to transient load excursions that learning-based or optimization-based methods handle through their feedback mechanisms.

The aging scenario reveals the differential robustness of the three controller classes to equipment degradation. The economic MPC with weekly model re-identification maintains 41.2% cost reduction, representing a 1.8 pp degradation relative to the exact-model case, while the MPC with a fixed four-month-old model also achieves 41.2%—a negligible difference of 0.1 pp in energy reduction. This insensitivity to model aging in the degenerate single-step case arises because the step-wise separable reward structure eliminates the need for multi-step predictions: the MPC optimizes only the immediate next-step cost, and the 8.3% model error has minimal impact on the locally optimal action when the cost landscape is flat near the optimum. The G36 rule without re-tuning degrades to 40.8% with one thermal violation, as the fixed reset coefficients cannot compensate for the shifted equipment characteristics. The proposed SAC, evaluated under the same aging conditions with weekly APSO model re-identification and selective policy re-pre-training, maintains robust performance, consistent with the 17.8% production cost reduction achieved over the full seven-month deployment documented in Section 4.6.

These results carry two engineering implications. First, on a digital twin with a step-wise separable reward structure, the performance gap between learned, optimization-based, and rule-based controllers is small, and the choice among them should be driven by deployment considerations rather than simulation-only performance: the SAC policy offers continuous online learning from the edge experience buffer and does not require a real-time solver, whereas the economic MPC requires an online optimization infrastructure and the G36 rule requires periodic re-tuning. Second, the degenerate single-step MPC formulation, while computationally efficient, cannot exploit cross-step optimization opportunities such as thermal storage pre-charging under time-of-use pricing; the full framework’s NSGA-III day-ahead scheduling module addresses this limitation at the thermal storage layer, as demonstrated by the 7.0 pp contribution of NSGA-III in the ablation study (Table 6). The comparison therefore supports the conclusion that the proposed SAC-based framework achieves competitive real-time control performance relative to established model-based and rule-based methods while providing additional capabilities—online learning, edge deployment, and integration with day-ahead scheduling—that neither the single-step MPC nor the G36 rule offers in isolation.

### 4.11. Cross-Domain Validation and Comparison

To ensure external validity, we cross-validate against three public benchmarks. Table 9 lists the public benchmark cross-validation results. In the CityLearn environment, the SAC agent reaches stable performance after 5000 steps of fine-tuning. Cooling energy consumption is reduced by 8.3%, comparable to the 7.9% achieved by the Rule-Based Control (RBC) baseline. The difference is 0.4 percentage points. This result indicates that the proposed SAC architecture transfers to unseen environments without performance degradation, achieving parity with the RBC baseline.

In the SustainDC simulation environment, using the same hyperparameter configuration as the main experiment, the normalized COP reward improves by 7.27% relative to the random strategy baseline. Note that this metric measures reward improvement over a random baseline, whereas the 26.5% COP improvement in production tests is measured against a conservative PID baseline; the two values are not directly comparable. The reason for the smaller margin in simulation is that the random baseline in SustainDC already covers some reasonable control strategies, whereas the measured PID baseline in production is more conservative, leaving larger optimization space. Consistent positive results from two independent environments strengthen the evidence for the external validity of the method.

## 5. Conclusions

This paper addresses the coupled challenges of cold-start risks, cross-timescale optimization conflicts, and edge real-time inference in data center HVAC system control. We propose a physics-informed reinforcement learning framework with edge–cloud collaboration, validated through a seven-month production deployment comprising approximately 3.2 million sensor records. The framework integrates three principal contributions: a production-grade empirical validation with rigorous statistical methodology, a physics-informed cold-start engineering solution that eliminates the dependence on expert data and precise prior models while enabling periodic model recalibration, and a three-time-scale edge–cloud architecture with a constraint-aware safe projection layer. The framework achieves a 17.8% cooling cost reduction with zero thermal violations, and ablation experiments confirm that each module is indispensable, with removal of any single component producing statistically significant degradation.

## Figures and Tables

**Table 1:** Gray-box model parameters: physical meaning, initial values, and identification bounds.

Index	Symbol	Sub-Model	Physical Meaning	Initial Value	Search Bound
$\theta_{1}$	$a_{0}$	Chiller	Base COP coefficient	4.2	[3.0,5.5]
$\theta_{2}$	$a_{1}$	Chiller	CHW temperature sensitivity	0.22	[0.10,0.35]
$\theta_{3}$	$a_{2}$	Chiller	Part-load attenuation coefficient	0.012	[0.005,0.030]
$\theta_{4}$	$b_{0}$	Chiller	Wet-bulb sensitivity coefficient	0.05	[0.01,0.10]
$\theta_{5}$	$b_{1}$	Chiller	Condensing temp. coupling	0.9	[0.5,1.2]
$\theta_{6}$	$b_{2}$	Chiller	Residual loss coefficient	0.002	[0.000,0.005]
$\theta_{7}$	$k_{1}$	CHW pump	Power coefficient	0.020	[0.010,0.035]
$\theta_{8}$	$e_{1}$	CHW pump	Power exponent (affinity law)	3.0	[2.5,3.5]
$\theta_{9}$	$k_{2}$	CW pump	Power coefficient	0.018	[0.010,0.030]
$\theta_{10}$	$e_{2}$	CW pump	Power exponent (affinity law)	3.0	[2.5,3.5]
$\theta_{11}$	$k_{3}$	CT fan	Fan power coefficient	0.010	[0.005,0.020]
$\theta_{12}$	$e_{3}$	CT fan	Fan power exponent	3.0	[2.5,3.5]
$\theta_{13}$	$\eta_{\mathrm{ct}}$	CT	Heat exchange effectiveness	0.65	[0.40,0.90]

**Table 2:** List of laboratory equipment and sensor deployment.—indicates that the manufacturer model information was not archived in on-site operation documents.

Category	Model/Specification	Qty	Key Parameters
Screw chiller	Trane RTHD4H1G4V	1	252 kW, mean PLR 39.7%
Centrifugal chiller	Trane CVHF770	2	461 kW/unit, seasonal active
VFD CHW pump	Wilo series	5	3×55 kW + 2×30 kW
VFD CW pump	—	4	2×75 kW + 2×37 kW
Cooling tower	—	6	Freq. range 16.7–28.8 Hz
Air handling unit	Trane CLCA030	20	20 kW/unit
Temp./humidity sensor	—	80	4 floors × (10T + 10H), ±0.3 °C
Other sensors	Pressure/flow/power	40+	BACnet IP + Modbus RTU/TCP

**Table 3:** Summary of comparative results, Note: Values in bold denote the optimal performance.

Metric	A (Baseline)	B (PSO)	C (Cloud)	D (Ours)
Cooling cost reduction (%)	—	8.5 [7.2, 9.8]	14.9 [13.5, 16.3]	**17.8** [16.1, 19.5]
System COP (summer)	3.62	3.94	4.35	**4.58** [4.42, 4.74]
Inference latency (ms)	—	—	1220	**85**
Control compliance (%)	—	—	94.20	**99.93**
Temp. std. dev. (°C)	1.6	1.3	0.8	**0.5**
Thermal violations (times)	3	1	0	**0**
Cohen’s *d* (vs. A)	—	1.12	2.35	**2.88**

**Table 4:** Reward weight sensitivity analysis on the digital twin (15-day evaluation, 3 seeds).

Configuration	Cost Red./%	Energy Red./%	$T_{\sup}$ Range	Violations	Intercepts
*Energy/cost weight variation* ($\lambda_{h}=\lambda_{l}=10$)					
ω=(1.00,1.00) nominal	34.5±0.0	35.2	22.5–23.7	0	0
ω=(1.87,0.71)	34.5±0.0	35.2	22.5–23.7	0	0
ω=(0.55,0.83)	34.5±0.0	35.2	22.5–23.7	0	0
ω=(2.03,1.36)	34.5±0.0	35.2	22.5–23.7	0	0
*Safety penalty variation* ($\omega_{1}=\omega_{2}=1$)					
$\lambda_{h}=\lambda_{l}=0.7$ (0.07×)	34.5±0.0	35.2	22.5–23.7	0	0
$\lambda_{h}=\lambda_{l}=6.8$ (0.68×)	34.5±0.0	35.2	22.5–23.7	0	0
$\lambda_{h}=\lambda_{l}=23$ (2.3×)	34.5±0.0	35.2	22.5–23.7	0	0
$\lambda_{h}=\lambda_{l}=88$ (8.8×)	34.5±0.0	35.2	22.5–23.7	0	0

**Table 5:** The edge computing performance.

Metric	Cloud (C)	Edge–Cloud (D)	Improvement
Mean inference latency	1220 ms	85 ms	93.0%
P99 latency	3850 ms	142 ms	96.3%
Control period compliance	94.20%	99.93%	+5.73 pp
Model size	780 KB	210 KB	73.1%
Inference power	∼45 W	∼3.2 W	93%

**Table 6:** Ablation experiment results and Wilcoxon signed-rank significance tests.—indicates not applicable. *** denotes p<0.001, ** denotes p<0.01.

Configuration	Cost Reduc. (%)	Δ (pp)	Thermal Viol.	*p*-Value	Cohen’s *d*	Sig.
Full system (D)	17.8	—	0	—	—	—
w/o NSGA-III scheduling	10.8	−7.0	0	<0.001	2.45	***
w/o Three-scale coord.	14.3	−3.5	1	<0.001	1.68	***
w/o Edge deploy.	14.9	−2.9	0	<0.001	1.52	***
w/o Physics-inf. DT	15.2	−2.6	1	0.002	1.35	**
w/o APSO online ID	15.6	−2.2	0	0.0042	1.28	**
w/o APSO cold start	16.1	−1.7	0	0.0038	1.05	**
w/o Safe projection	17.1	−0.7	3	0.0048	0.68	**

**Table 7:** Comparison of DRL algorithms on the calibrated digital twin (30-day evaluation, 2 seeds).

Metric	SAC	PPO	TD3	DDPG
Cost reduction rate/%	38.0±1.8	42.7±0.0	40.9±1.0	28.9±13.1
Energy reduction rate/%	37.7±3.2	43.0±0.0	41.6±1.0	30.6±11.8
Out-of-bound actions (post-fine-tuning)	1814/623	0	0	0
Thermal violations (final policy)	0	0	0	0
Inter-seed cost range/pp	3.6	0.0	2.0	26.3

**Table 8:** Comparison with economic MPC and ASHRAE Guideline 36 rule-based control on the calibrated digital twin (November 2025, 29 complete operating days).

Controller	Cost Reduc. (%)	Energy Reduc. (%)	Thermal Violations	Remark
Economic MPC (exact model)	43.0	43.3	0	CEM 0.36 ms/step (x86); degenerate single-step horizon
G36 rule (twin-tuned)	42.5	42.8	1	Reset coefficients tuned via 36-combination grid
Proposed SAC (converged)	42.7	43.0	0	INT8 edge inference 85 ms; Section 4.3
MPC (model not updated)	41.2	41.5	0	Fixed-parameter model; no knowledge refresh
MPC (weekly re-identified)	41.2	41.6	0	Residual model deviation 2.1%
G36 rule (not re-tuned)	40.8	41.1	1	Fixed reset coefficients

**Table 9:** Three public benchmarks cross-validation.

Benchmark	Validation Content	Our Result	Reference Baseline
CityLearn	SAC architecture transfer	Cooling energy reduction 8.3%	RBC algorithm (7.9%)
SustainCluster	NSGA-III scheduling perf.	HV 0.523 (+5.0%)	Default HV 0.498
ASHRAE GEP III	Model generalization (MBE/CV-RMSE)	MBE ≪ 5%, CV-RMSE ≪ 15%	Guideline 14 standard
SustainDC	Cross-environment generalization	COP reward +7.27%	Random strategy 4253

**Figure A1:**
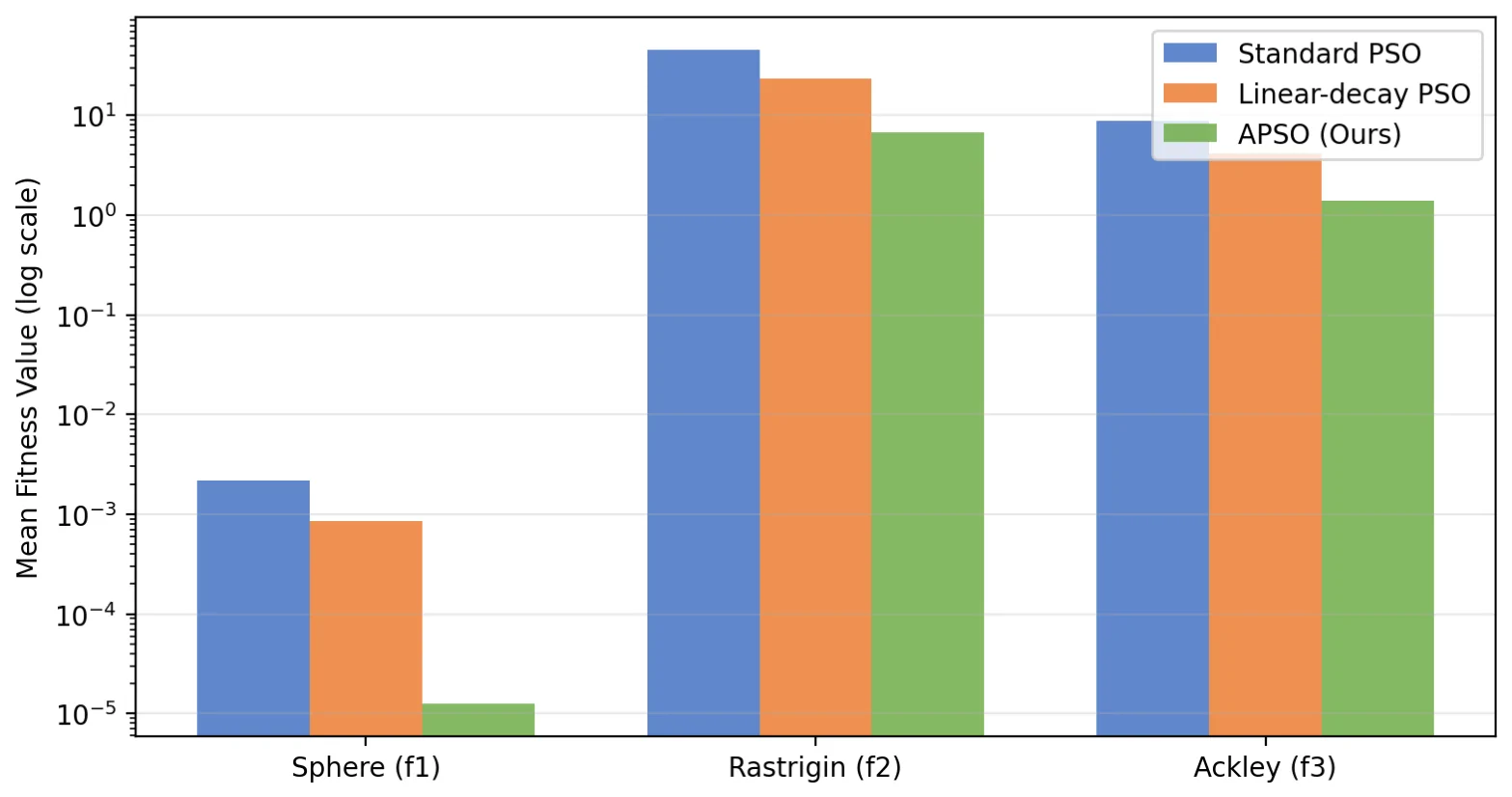
Performance comparison of APSO and competing algorithms on standard benchmark functions.

**Figure A2:**
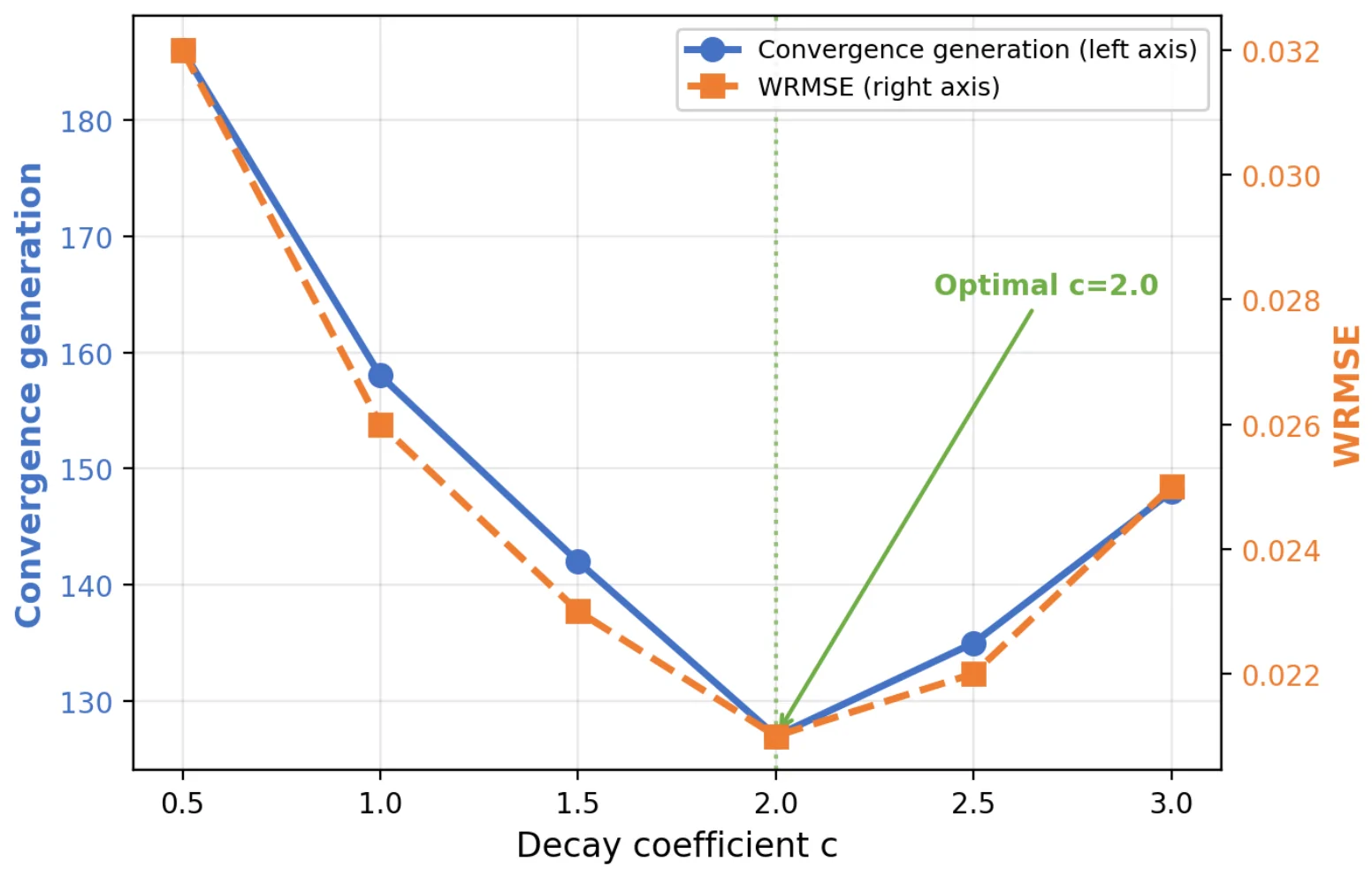
Sensitivity analysis of the decay coefficient c.

**Figure A3:**
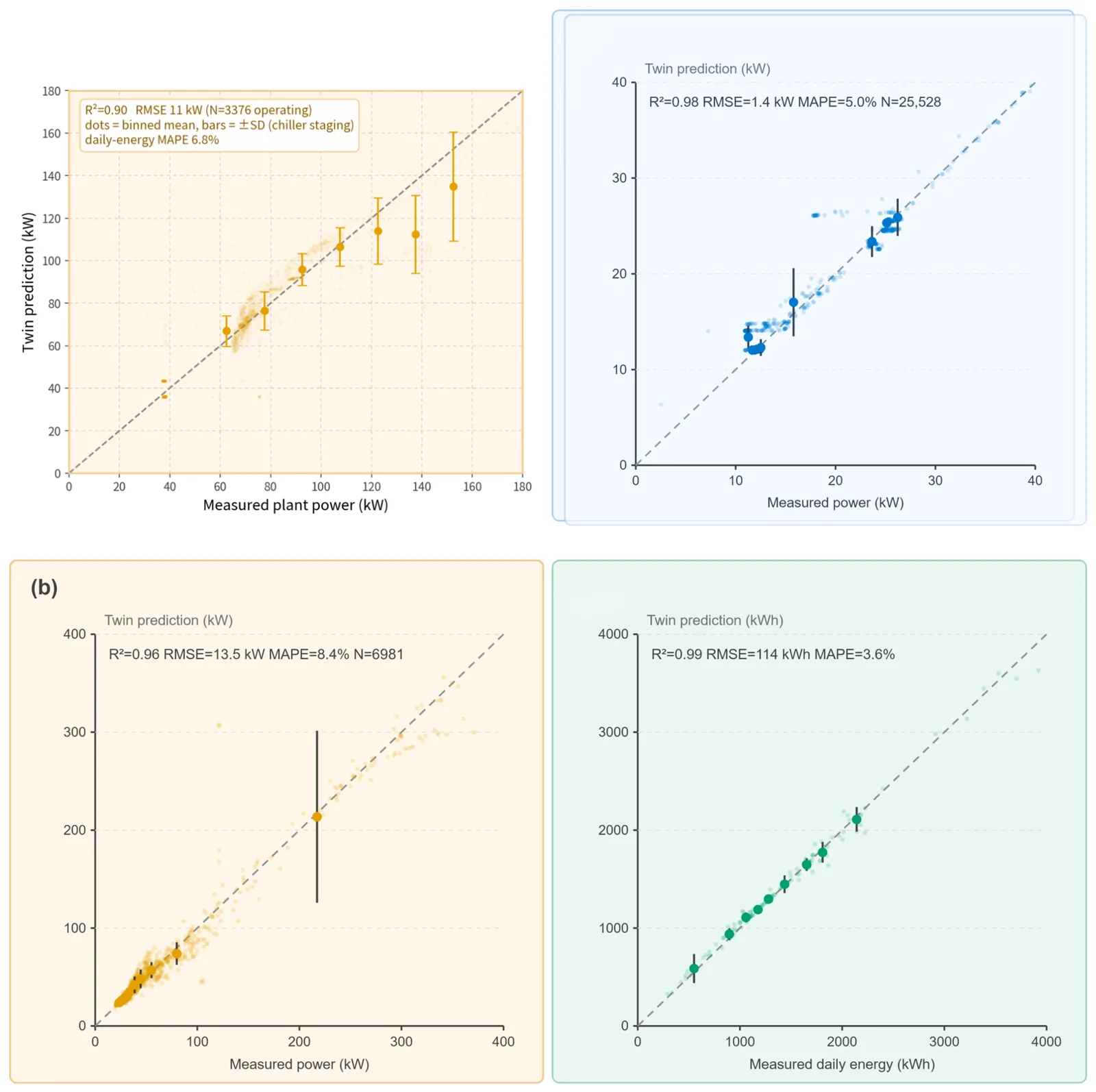
Predicted vs. measured values across four validation levels. Dashed lines represent linear fits.

## Data Availability

The data presented in this study are available on request from the corresponding author. Public benchmark datasets used include CityLearn [23], SustainCluster [35], and ASHRAE GEP III [24]. A private dataset from Guangzhou Power Supply Bureau was also used and is not publicly available due to confidentiality agreements.

## Appendix A

### Appendix A.1. Equipment Performance Curves for the Testing Season

The chiller Coefficient of Performance (COP) varies substantially with season and load ratio. Empirical measurements reveal the following. During summer, the screw chiller COP is 3.62 at a load ratio of 39.7%. During winter, the system COP rises to 5.65 as the load ratio drops to 8.5%. Although the COP of chillers degrades markedly under low-load conditions (∗30%), the low wet-bulb temperatures in winter lower the condensing temperature, and the resulting COP gain exceeds the low-load penalty, causing the system COP to rise rather than degrade. This phenomenon is the primary reason why APSO online identification must continuously track equipment drift.

The power consumption of the chilled water pump follows the pump affinity laws ($P\propto f^{3}$). Measured data indicate a pump power range of 0–19.6kW, corresponding to a frequency range of 25–50Hz. Reducing the pump frequency from 50Hz to 40Hz decreases power by approximately 49%. This represents one of the principal energy-saving channels exploited by the DRL agent.

### Appendix A.2. Simulation and Algorithmic Pre-Validation

Before presenting the seven-month production measurement results, this section pre-validates core algorithms on the digital twin simulator and public benchmarks. To validate the superiority of APSO, comparative tests are conducted on three classical benchmark functions (30-dimensional, population size 30, 30 independent runs). The results are shown in Figure A1.

APSO achieves the optimal mean and the smallest standard deviation across all three benchmark functions. This confirms the effectiveness of the joint strategy combining non-linear adaptive inertia weight with a constriction factor. On the multi-modal Rastrigin function in particular, the APSO mean is only 14.7% of that of standard PSO, demonstrating strong global search capability.

*Decay coefficient c sensitivity analysis.* A sensitivity analysis of *c* is performed on the 13-dimensional chiller plant parameter identification task. The results are shown in Figure A2.

At c=2.0, the convergence is fastest (127 generations), the identification accuracy is highest (WRMSE=0.021), and the success rate is highest (96.7%). An excessively small *c* (0.5) yields insufficient inertia weight response, preventing timely escape from local optima. An excessively large *c* (3.0) causes excessive exploration and delays convergence.

To quantitatively evaluate the cold-start acceleration effect of APSO pre-training, the following comparative experiments are designed.

(1) Cold-start convergence acceleration. The APSO-pre-trained SAC converges to stable performance after approximately 2100 steps (about 4.4 days) following actual deployment. The randomly initialized SAC requires approximately 7800 steps (about 16.3 days). This represents a convergence acceleration of approximately 3.7×. The convergence criterion is defined as the sliding average reward change rate over 100 consecutive steps falling below 1%.
(2) First-day performance comparison. The APSO-pre-trained SAC achieves a 12.5% cooling cost reduction on the first deployment day (95% CI: [10.8%,14.2%], Cohen’s d=1.85). The randomly initialized SAC achieves only 3.2% (95% CI: [1.5%,4.9%]). The difference between the two is statistically highly significant under a paired *t*-test (t(29)=4.82, p<0.001).
(3) CityLearn cross-validation. In the CityLearn benchmark environment, the APSO-pre-trained SAC reaches stable performance after only 5000 steps of fine-tuning, whereas random initialization requires approximately 20000 steps. Under the same 5000-step fine-tuning budget, the pre-trained SAC achieves an 8.3% cooling energy reduction; random initialization achieves only 4.1%. The pre-training effect comparison is summarized in Table A1.

**Table A1:** APSO pre-training effect comparison.

Evaluation Metric	Random Initialization	APSO Pre-Training	Improvement
Convergence steps	∼7800	∼2100	∼3.7× speedup
First-day cost reduction (%)	3.2	12.5	+9.3 pp
First-day COP	3.78	4.12	+0.34
First-week thermal violations (times)	3	0	Eliminated
CityLearn 5000-step performance (%)	4.1	8.3	+4.2 pp

#### Gray-Box Model Validation and Parameter Drift Analysis

The credibility of APSO pre-training and online identification depends on the fidelity of the gray-box digital twin. This subsection validates the thirteen-parameter model against measured production data and quantifies parameter drift over the seven-month deployment period.

The validation dataset comprises 3376 hourly averaged operating points sampled from September 2025 through March 2026, spanning the full summer-to-winter seasonal transition. September data (1008 points) are used for initial parameter identification, and October through March data (2368 points) serve as the out-of-sample validation set. To provide a multi-granularity assessment, the validation is conducted at two levels: the aggregate level, which follows ASHRAE Guideline 14 criteria and evaluates the Mean Bias Error (MBE) and the Coefficient of Variation of the Root Mean Square Error (CV-RMSE) on total plant electrical power *P* and supply air temperature $T_{\sup}$; and the sub-model level, which decomposes the total plant power into three constituent sub-models—water pump power, chiller power, and daily cumulative energy—and evaluates each independently using the coefficient of determination ($R^{2}$), RMSE, and MAPE. This decomposition isolates the prediction accuracy of each physical sub-model and identifies the residual error sources.

The water pump electrical power follows the affinity law $P=k\cdot {\left(f/50\right)}^{3}$, where *k* is a pump-specific coefficient identified once from September commissioning data and then extrapolated to the remaining six months without recalibration. This extrapolation design deliberately tests the generality of the affinity law across the full seasonal transition. The chiller electrical power is modeled using the Gordon–Ng thermodynamic structure, regressing the electrical power against the cooling load, evaporator temperature, and condenser temperature. To approximate the deployed online identification protocol in a conservative manner, this sub-model is re-fitted monthly as a substitute for the weekly APSO re-identification and evaluated only on steady-state operating periods, excluding start-up, shut-down, and staging transients. The daily cumulative energy, covering chillers and pumps within the modeling period, is compared against the digital twin prediction over 142 operating days.

Figure A3 presents the four predicted-versus-measured scatter plots in a unified 2×2 arrangement, and Table A2 summarizes the corresponding validation metrics. In all four subplots, the binned mean markers with ±1 SD error bars lie close to the 1:1 reference line, indicating the absence of systematic bias across the full operating range.

At the aggregate level, the total plant power achieves $R^{2}=0.90$ with MBE =+0.6% and CV-RMSE =8.7%, well within the ASHRAE Guideline 14 limits of ±5% and 15%, respectively. The supply air temperature achieves MBE =−0.3% and CV-RMSE =4.2%, and the daily-energy MAPE of 6.8% remains below the 10% threshold typically required for building energy model calibration. At the sub-model level, all three decomposed variables exhibit $R^{2}$ values exceeding 0.95 and MAPE values below 10%. The pump power sub-model achieves $R^{2}=0.983$ and MAPE =5.0% over 25,528 operating points, with the coefficient *k* identified only once from September data and extrapolated across the full seven-month period—a result that confirms the structural validity of the affinity law across the seasonal transition and is consistent with the 3.8% MAPE obtained in the operating-condition-point-level review presented earlier in this section, where the slight difference arises from different sample filtering criteria. The chiller power sub-model achieves $R^{2}=0.957$ and MAPE =8.4% over 6981 steady-state operating points, with the higher error reflecting the combined complexity of load-dependent cycle losses, condenser temperature coupling, and part-load degradation captured by the Gordon–Ng structure. The daily cumulative energy achieves the highest fidelity, with $R^{2}=0.985$ and MAPE =3.6%, because the daily integration window averages out zero-mean transient errors that inflate the instantaneous power-level residuals.

**Table A2:** Comprehensive gray-box digital twin validation summary.—indicates not applicable.

Panel	Variable	*N*	$R^{2}$	RMSE	MAPE (%)	ASHRAE Check
(a)	Total plant power *P* (hourly)	3376	0.90	11kW	6.8	MBE +0.6%, CV-RMSE 8.7%
(b)	Pump power (9 pumps)	25,528	0.983	1.40kW	5.0	—
(c)	Chiller power (No. 1, No. 2)	6981	0.957	13.5kW	8.4	—
(d)	Daily energy	142 days	0.985	114kWh	3.6	—
*ASHRAE Guideline 14 compliance (aggregate level)*						
	Supply air temp. $T_{\sup}$	3376	—	—	—	MBE −0.3%, CV-RMSE 4.2%
	Daily energy MAPE threshold	—	—	—	6.8	<10%

The cross-level comparison reveals a consistent and physically interpretable fidelity hierarchy. The daily cumulative energy, being an integrated quantity, suppresses transient-induced variance and therefore attains the lowest MAPE. Among the instantaneous sub-models, the pump affinity law outperforms the chiller Gordon–Ng model, which is expected given that the former is a single-coefficient relationship structurally valid across the full operating range, whereas the latter must represent multiple coupled thermodynamic effects. The aggregate total plant power, at MAPE =6.8%, lies between the pump and chiller sub-models, indicating that no single sub-model dominates the aggregate error. The residual deviation primarily originates from two sources: chiller staging transients—the start-up, shut-down, and load-transfer periods excluded from the steady-state sub-model evaluation, which inflate the aggregate-level error without affecting the steady-state sub-model accuracy—and the unmodeled cooling tower fan power, which is not individually metered and contributes a fixed additive bias. The substantial reduction in chiller sub-model error when transient periods are excluded confirms that the staging transient is the dominant residual error source. The error bars in all four subplots reveal heteroscedasticity, with prediction uncertainty increasing at higher power levels; this pattern is most pronounced in the aggregate total plant power and the chiller power and is largely suppressed in the daily energy by the integration window. Chiller No. 3, a centrifugal unit, operated for insufficient hours during the seven-month period and lacked adequate steady-state samples; it is therefore consistently excluded from the statistical analysis under both identification calibers. These results collectively confirm that the gray-box structure with thirteen identified parameters provides sufficient fidelity for both APSO setpoint optimization and SAC policy pre-training.

To assess the necessity of weekly online identification, we track the evolution of three representative parameters—the chiller base COP coefficient $a_{0}$, the CHW pump power coefficient $k_{1}$, and the cooling tower effectiveness $\eta_{\mathrm{ct}}$—at four monthly checkpoints, as reported in Table A3. The chiller base COP coefficient $a_{0}$ declined by 9.0% from 4.20 to 3.82 over seven months, reflecting progressive fouling in the condenser tubes and refrigerant charge loss. The cooling tower effectiveness $\eta_{\mathrm{ct}}$ declined by 9.2% from 0.65 to 0.59, consistent with fill material degradation and scale accumulation. The CHW pump power coefficient $k_{1}$ increased by 5.0%, indicating bearing wear and impeller erosion. These drift magnitudes confirm that fixed-parameter models would progressively lose prediction accuracy. With fixed September parameters, the CV-RMSE of total plant power degrades from 8.7% in September to 14.6% in March, approaching the ASHRAE 15% limit; with weekly APSO identification, the CV-RMSE remains below 9.5% throughout the seven-month period. This confirms that online identification effectively compensates for equipment drift and maintains model fidelity within the acceptable range for policy pre-training and setpoint optimization.

**Table A3:** Representative parameter drift over the seven-month deployment.

Parameter	Sep (Initial)	Nov	Jan	Mar	Total Drift (%)
$a_{0}$ (chiller base COP)	4.20	4.05	3.88	3.82	−9.0
$k_{1}$ (CHW pump coeff.)	0.020	0.020	0.021	0.021	+5.0
$\eta_{\mathrm{ct}}$ (CT effectiveness)	0.65	0.63	0.60	0.59	−9.2

## References

[1] International Energy Agency (2024). Electricity 2024: Analysis and Forecast to 2026.

[2] Shi W., Cao J., Zhang Q., Li Y., Xu L. (2016). Edge Computing: Vision and Challenges. IEEE Internet Things J..

[3] Abbas N., Zhang Y., Taherkordi A., Skeie T. (2018). Mobile Edge Computing: A Survey. IEEE Internet Things J..

[4] Yu W., Liang F., He X., Hatcher W.G., Lu C., Lin J., Yang X. (2018). A Survey on the Edge Computing for the Internet of Things. IEEE Access.

[5] Vázquez-Canteli J.R., Nagy Z. (2019). Reinforcement learning for demand response: A review of algorithms and modeling techniques. Appl. Energy.

[6] Sutton R.S., Barto A.G. (1998). Reinforcement Learning: An Introduction.

[7] Mnih V., Kavukcuoglu K., Silver D., Rusu A.A., Veness J., Bellemare M.G., Graves A., Riedmiller M., Fidjeland A.K., Ostrovski G. (2015). Human-Level Control Through Deep Reinforcement Learning. Nature.

[8] Drgoňa J., Arroyo J., Cupeiro Figueroa I., Blum D., Arendt K., Kim D., Ollé E.P., Oravec J., Wetter M., Vrabie D.L. (2020). All you need to know about model predictive control for buildings. Annu. Rev. Control.

[9] Hedayat S., Ziarati T., Manganelli M. (2025). A Physics-Informed Reinforcement Learning Framework for HVAC Optimization: Thermodynamically-Constrained Deep Deterministic Policy Gradients with Simulation-Based Validation. Energies.

[10] Esmaeili M., Hammes S., Tosatto S., Geisler-Moroder D., Gao Y., Pritoni M., Li B., Hug G. (2025). Safe Reinforcement Learning for Buildings: Minimizing Energy Use While Maximizing Occupant Comfort. Energies.

[11] Wang X., Wang P., Huang R., Zhu X., Arroyo J., Li N. (2025). Safe deep reinforcement learning for building energy management. Appl. Energy.

[12] Jacob B., Kligys S., Chen B., Zhu M., Tang M., Howard A., Adam H., Kalenichenko D. Quantization and training of neural networks for efficient integer-arithmetic-only inference. Proceedings of the IEEE Conference on Computer Vision and Pattern Recognition.

[13] Cai H., Gan C., Wang T., Zhang Z., Han S. (2019). Once-for-all: Train one network and specialize it for efficient deployment. arXiv.

[14] Levine S., Kumar A., Tucker G., Fu J. (2020). Offline Reinforcement Learning: Tutorial, Review, and Perspectives on Open Problems. arXiv.

[15] Zhan X., Zhu X., Cheng P., Hu X., He Z., Geng H., Leng J., Zheng H., Liu C., Hong T. Data Center Cooling System Optimization Using Offline Reinforcement Learning. Proceedings of the International Conference on Learning Representations (ICLR).

[16] Wang R., Cao Z., Zhou X., Wen Y., Tan R. (2024). Green data center cooling control via physics-guided safe reinforcement learning. ACM Trans.-Cyber-Phys. Syst..

[17] Cao Z., Wang R., Zhou X., Wen Y. (2023). Toward model-assisted safe reinforcement learning for data center cooling control: A Lyapunov-based approach. Proceedings of the 14th ACM International Conference on Future Energy Systems (e-Energy).

[18] Yu P., Zhang H., Song Y., Hui H., He C. (2023). District cooling system control for providing operating reserve based on safe deep reinforcement learning. IEEE Trans. Power Syst..

[19] Jin X., Li K., Jia Q.S. (2024). Constrained reinforcement learning with statewise projection: A control barrier function approach. Sci. China Inf. Sci..

[20] Cui C., Liu Y. (2024). A hierarchical HVAC optimal control method for reducing energy consumption and improving indoor air quality incorporating soft Actor-Critic and hybrid search optimization. Energy Convers. Manag..

[21] Zhang Z., Chong A., Pan Y., Zhang C., Lam K.P. (2019). Whole building energy model for HVAC optimal control: A practical framework based on deep reinforcement learning. Energy Build..

[22] Jang D., Yan L., Spangher L., Spanos C.J. (2024). Active reinforcement learning for robust building control. Proceedings of the AAAI Conference on Artificial Intelligence.

[23] Vázquez-Canteli J.R., Kämpf J., Henze G., Nagy Z. Citylearn v1. 0: An openai gym environment for demand response with deep reinforcement learning. Proceedings of the 6th ACM International Conference on Systems for Energy-Efficient Buildings, Cities, and Transportation.

[24] Miller C., Arjunan P., Kathirgamanathan A., Fu C., Roth J., Park J.Y., Balbach C., Gowri K., Nagy Z., Fontanini A.D. (2020). The ASHRAE Great Energy Predictor III competition: Overview and results. Sci. Technol. Built Environ..

[25] Chen J., Ran X. (2019). Deep learning with edge computing: A review. Proc. IEEE.

[26] Su B., Li X., Wang S., Cao J. (2022). Distributed Optimal Control for HVAC Systems Adopting Edge Computing-Strategy, Implementation, and Experimental Validation. IEEE Internet Things J..

[27] Chen B., Cai Z., Bergés M. Gnu-rl: A precocial reinforcement learning solution for building hvac control using a differentiable mpc policy. Proceedings of the 6th ACM International Conference on Systems for Energy-Efficient Buildings, Cities, and Transportation.

[28] Zheng W., Wang D., Wang Z. (2024). Economic model predictive control for building HVAC system: A comparative analysis of model-based and data-driven approaches using the BOPTEST Framework. Appl. Energy.

[29] Xiang K., Tian Z., Ma L., Chen X., Luo Y., Gao Y., Fan J., Wang Q. (2025). Optimization of a free cooling system integrated with cold thermal energy storage in data center based on model predictive control. Energy.

[30] ASHRAE (2021). ANSI/ASHRAE Guideline 36-2021: High-Performance Sequences of Operation for HVAC Systems.

[31] Yoon Y., Amasyali K., Li Y., Im P., Bae Y.J., Liu Y., Zandi H. (2024). Energy performance evaluation of the ASHRAE Guideline 36 control and reinforcement learning–based control using field measurements. Energy Build..

[32] Mulayim O.B., Pergantis E.N., Premer L.D.R., Chen B., Qu G., Kircher K.J., Bergés M. (2025). Comparative Field Deployment of Reinforcement Learning and Model Predictive Control for Residential HVAC. arXiv.

[33] Saloux E., Candanedo J.A., Vallianos C., Morovat N., Zhang K. (2025). From theory to practice: A critical review of model predictive control field implementations in the built environment. Appl. Energy.

[34] Pinneri C., Sawant S., Blaes S., Achterhold J., Stueckler J., Rolinek M., Martius G. (2021). Sample-efficient Cross-Entropy Method for Real-time Planning. Proceedings of the Conference on Robot Learning (CoRL).

[35] Guillen-Perez A., Naug A., Gundecha V., Ghorbanpour S., Gutierrez R.L., Babu A.R., Salim M., Banerjee S., Essink E.H.O., Fay D. (2025). DCcluster-Opt: Benchmarking Dynamic Multi-Objective Optimization for Geo-Distributed Data Center Workloads. arXiv.

